# Expression of potato RNA-binding proteins StUBA2a/b and StUBA2c induces hypersensitive-like cell death and early leaf senescence in *Arabidopsis*

**DOI:** 10.1093/jxb/erv207

**Published:** 2015-05-05

**Authors:** Jong-Kuk Na, Jae-Kwang Kim, Dool-Yi Kim, Sarah M. Assmann

**Affiliations:** ^1^Biology Department, Pennsylvania State University, University Park, Pennsylvania 16802USA; ^2^Division of Life Sciences, College of Life Sciences and Bioengineering, Incheon National University, Incheon 406-772, Republic of Korea; ^3^Crop Function Division, National Institute of Crop Science, Rural Development Administration, Wanju-gun, Jeollabuk-do 565-851, Republic of Korea; ^4^Molecular Breeding Division, National Academy of Agricultural Science, RDA, Wanju-gun, Jeollabuk-do 565-851, Republic of Korea

**Keywords:** AKIP proteins, *Arabidopsis thaliana*, RNA binding proteins, senescence, *Solanum tuberosum*, UBA2 proteins.

## Abstract

The *Arabidopsis thaliana* genome encodes three RNA-binding proteins (RBPs), UBP1-associated protein 2a (UBA2a), UBA2b, and UBA2c, that contain two RNA-recognition motif (RRM) domains. They play important roles in wounding response and leaf senescence, and are homologs of *Vicia faba* abscisic-acid-activated protein kinase-interacting protein 1 (VfAKIP1). The potato (*Solanum tuberosum*) genome encodes at least seven AKIP1-like RBPs. Here, two potato RBPs have been characterized, StUBA2a/b and StUBA2c, that are homologous to VfAKIP1 and *Arabidopsis* UBA2s. Transient expression of *StUBA2*s induced a hypersensitive-like cell death phenotype in tobacco leaves, and an RRM-domain deletion assay of StUBA2s revealed that the first RRM domain is crucial for the phenotype. Unlike overexpression of *Arabidopsis UBA2*s, constitutive expression of *StUBA2a/b* in *Arabidopsis* did not cause growth arrest and lethality at the young seedling stage, but induced early leaf senescence. This phenotype was associated with increased expression of defence- and senescence-associated genes, including pathogen-related genes (*PR*) and a senescence-associated gene (*SAG13*), and it was aggravated upon flowering and ultimately resulted in a shortened life cycle. Leaf senescence of *StUBA2a/b Arabidopsis* plants was enhanced under darkness and was accompanied by H_2_O_2_ accumulation and altered expression of autophagy-associated genes, which likely cause cellular damage and are proximate causes of the early leaf senescence. Expression of salicylic acid signalling and biosynthetic genes was also upregulated in *StUBA2a/b* plants. Consistent with the localization of UBA2s-GFPs and VfAKIP1-GFP, soluble-modified GFP-StUBA2s localized in the nucleus within nuclear speckles. StUBA2s potentially can be considered for transgenic approaches to induce potato shoot senescence, which is desirable at harvest.

## Highlight

Potato StUBA2 RNA-binding proteins promote hypersensitive-like cell death (dependent on the first StUBA2 RNA recognition motif) and premature leaf senescence, and increase transcripts of select pathogen-associated, senescence-associated, and autophagy-associated genes.

## Introduction

Plant development is achieved by transcriptional, post-transcriptional, and translational regulation of gene expression. Upstream regulatory regions play important roles in the initial transcription of protein-coding genes upon perception of cues, and subsequent post-transcriptional processes exert pivotal roles in modulating expression of specific transcripts through pre-mRNA splicing, alternative splicing, capping, polyadenylation, mRNA stability, and mRNA transport (Reddy *et al.*, 2012). RNA-binding proteins (RBPs) play crucial roles in such processes.

The genome of the model plant *Arabidopsis thaliana* contains ~200 RBPs, some of which are involved in stress response, plant immunity, or development (Lorkovic, 2009; Woloshen *et al.*, 2011). Many *Arabidopsis* RBPs are specific to plants, suggesting that these RBPs may have plant-specific functions (Lorkovic, 2009). Recent studies revealed roles of RBPs not only in various developmental processes such as floral development (Lim *et al.*, 2004; Mockler *et al.*, 2004; Streitner *et al.*, 2008), but also in responses to diverse environmental stresses such as abscisic acid (ABA) (Li *et al.*, 2002; Ng *et al.*, 2004; Bove *et al.*, 2008; Kim *et al.*, 2008), wounding (Bove *et al.*, 2008), cold stress responses (Kim *et al.*, 2010a; Kim *et al.*, 2010b), chromatin modification (Liu *et al.*, 2007; Baurle and Dean, 2008), leaf senescence (Kim *et al.*, 2008), and plant immunity (Woloshen *et al.*, 2011).

In *Vicia faba,* a guard-cell-specific ABA-activated serine-threonine protein kinase (AAPK) is integral in stomatal closure in response to ABA (Li *et al.*, 2000) and its orthologue in *Arabidopsis*, OST1, has been shown to be a central and limiting element in ABA signal transduction from soluble ABA receptors (Acharya *et al.*, 2013). Expression library screening identified AAPK interacting protein 1 (VfAKIP1), and phosphorylation of VfAKIP1 by AAPK is required for the interaction of VfAKIP1 with a target mRNA, *dehydrin*, in *V. faba* (Li *et al.*, 2002). In *Arabidopsis*, three UBP1-associated protein 2 (UBA2) proteins with two RNA-recognition motif (RRM) domains, UBA2a, UBA2b, and UBA2c, are homologous to VfAKIP1. UBA2a was previously identified from an interaction screen with the heterogeneous nuclear ribonucleoprotein (hnRNP) UBP1 protein, hence the designation ‘UBP1-associated protein 2’ (Lambermon *et al.*, 2002). VfAKIP1, UBA2a, and UBA2b fused with reporter GFP change their localization from a diffuse nuclear pattern to localization in nuclear speckles upon external ABA application (Li *et al.*, 2002; Ng *et al.*, 2004; Riera *et al.*, 2006; Bove *et al.*, 2008), while UBA2c forms nuclear speckles without ABA application (Bove *et al.*, 2008). Transient expression of each *Arabidopsis* UBA2 protein in *Nicotiana benthamiana* leaves induces a programmed-cell-death/senescing phenotype, while constitutive expression of each of these proteins causes an early lethality phenotype in *Arabidopsis* (Kim *et al.*, 2008). Controlled expression of the three UBA2s under a dexamethasone-inducible system showed that elevated expression of each of the three UBA2s causes leaf senescence (Kim *et al.*, 2008), indicating that UBA2s are positive regulators of leaf senescence. Recent work also revealed that *Arabidopsis* LAM-domain RBPs, LARP1b and LARP1c, are also positive regulators of leaf senescence. Overexpression of LARP1s induced leaf senescence in *Arabidopsis* and elevated transcript abundance of senescence- and defence-related genes, including senescence-associated genes (*SAGs*) and pathogen-related genes (*PR*s) (Zhang *et al.*, 2012). Both LARP1c and UBA2s are involved in leaf senescence seemingly as positive regulators, but they differ in subcellular localization with LARP1c found in the cytoplasm and UBA2s in the nucleus. Although underlying mechanisms for how UBA2s induce plant leaf senescence are not well defined, it is obvious that these RBPs play crucial roles in plant leaf senescence as positive regulators.

Plant senescence occurs as the final developmental stage, leading to the death of part or all of the plant (Lim *et al.*, 2007; Zhang and Zhou, 2012). Leaf yellowing, caused by chlorophyll loss, is one of the key indicators of the progression of leaf senescence, followed by programmed cell death. Leaf senescence is accompanied by cellular, biochemical, and molecular changes. At the molecular level, *SAGs* and defence-associated *PR* genes are upregulated with progression of senescence (Lim *et al.*, 2007). Consistently, overexpression of *LARP1c* or *Arabidopsis UBA2s* in *Arabidopsis* also increases the expression of *SAG* and *PR* genes (Kim *et al.*, 2008; Zhang *et al.*, 2012). Leaf senescence is also triggered by plant hormones such as ethylene, jasmonic acid, ABA, and salicylic acid (SA), and can be repressed by cytokinin (Zhang and Zhou, 2012). *Arabidopsis* UBA2 overexpression induces ethylene accumulation (Kim *et al.*, 2008), implying that ethylene accumulation in UBA2 overexpressors may promote or cause leaf senescence. SA accumulation also can induce not only hypersensitive-like cell death, but also the expression of *SAG* (Buchanan-Wollaston *et al.*, 2005; Zhang and Zhou, 2012) and *PR* genes (Durrant and Dong, 2004; Zhang *et al.*, 2010). SA is closely related to the control of levels of reactive oxygen species (ROS); ROS are upstream of SA signalling pathways but SA accumulation can induce ROS accumulation by feedback amplification (Petrov and Van Breusegem, 2012). The cell death phenotypes of UBA2s and LARP1c transgenic *Arabidopsis* plants might also result from elevated SA and/or ROS content, which can mediate the hypersensitive response of plants and ultimately lead to leaf senescence and cell death.

Here, the potato RBPs StUBA2a/b and StUBA2c are characterized and shown to have high homology in amino acid sequence to VfAKIP1 and *Arabidopsis* UBA2s. StUBA2a/b and StUBA2c were identified *in silico* by sequence homology to VfAKIP1 and *Arabidopsis* UBA2s and, like VfAKIP1 and UBA2s, contain two conserved RRMs. Transient expression of either of *StUBA2a/b* or *StUBA2c* induced a hypersensitive-like cell death phenotype in tobacco (*Nicotiana tabacum*) leaves, and the stable overexpression of *StUBA2a/b* in *Arabidopsis* led to early leaf senescence and ROS accumulation, and altered the expression of genes involved in SA production and autophagy. Taken together, these results suggest that StUBA2a/b and StUBA2c play a crucial role in leaf senescence.

## Materials and methods

### Plant materials and growth conditions

*Arabidopsis thaliana* ecotype Columbia (Col-0) was used in this study. Seeds were surface-sterilized and planted on half-strength Murashige and Skoog (MS) medium (Sigma) (Murashige and Skoog, 1962). After stratification for 3 days at 4°C, plates with seeds were transferred to a growth chamber (Controlled Environments Ltd.) and cultured for 2 weeks under short-day light conditions (8h of light, 20°C/16h dark, 18°C) at a light intensity of 120 µmol m^−2^ s^−1^. Then, seedlings were transferred to 16cm^2^ square pots filled with Miracle-Grow potting mix (Scotts) supplemented with perlite and grown under long-day light conditions (16h of light, 20°C/8h of dark, 18°C) at a light intensity of 120 µmol m^−2^ s^−1^ and 60–70% relative humidity.

### Cloning of potato StUBA2a/b and StUBA2c

Amino acid sequences of RBPs VfAKIP1 (Li *et al.*, 2000) and *Arabidopsis* UBA2s (Bove *et al.*, 2008; Kim *et al.*, 2008) were used to identify homologous EST sequences from the TIGR potato EST database (http://plantta.jcvi.org) using the Basic Local Alignment Search Tool (BLAST). Two *VfAKIP1-like* EST sequences were obtained from the TIGR database, and these EST sequences were used to inform cloning of *VfAKIP1-like* cDNAs from *S. tuberosum* ‘Atlantic’. Atlantic potato was obtained from the potato Germplasm Center (US Potato Genebank) and was aseptically grown in Magenta boxes for a month, followed by extraction of total RNA. cDNA synthesized from total RNA using Superscript III (Invitrogen) was used for PCR amplification of 1577bp *StUBA2a/b* and 1349bp *StUBA2c* using *StUBA2a/b* or *StUBA2c* gene-specific primer sets (Supplementary Table S1). PCR fragments of *StUBA2a/b* or *StUBA2c* were cloned into PCR-Blunt II-TOPO cloning vectors (Invitrogen) and sequenced. The amino acid sequence of StUBA2c was identical to that in the TIGR database and also that in the protein database released by the Potato Genome Sequencing Consortium (Xu *et al.*, 2011), but StUBA2a/b showed three and seven amino acid differences compared to that in the TIGR database or potato genome protein database, likely due to single nucleotide polymorphisms among cultivars. Subsequently, SacI/XbaI digests of *StUBA2a/b* or *StUBA2c* were subcloned into SacI/SpeI sites of the modified pORE-R2 binary vector (Coutu *et al.*, 2007) harbouring the *CaMV 35S* promoter to generate *35S:StUBA2a/b* and *35S:StUBA2c* constructs. RRM-deleted *StUBA2a/b* and *StUBA2c* were generated from pORE-R2 binary vectors harbouring *35S:StUBA2a/b* or *35S:StUBA2c* cassettes using gene-specific primers (Supplementary Table S1) by following the mutagenesis method described for the In-Fusion HD cloning system (Clontech Laboratories, Inc.). To generate the modified pORE-R2 with *CaMV 35S* promoter, a HindIII/XbaI fragment containing the *CaMV 35S* promoter from pGWB8 binary vector (Nakagawa *et al.*, 2007) was cloned into the HindIII/XbaI site of the pORE-R2 vector (Coutu *et al.*, 2007).

### Constructs of guard-cell-specific *pGC1:smGFP-StUBA2a/b* and *-StUBA2c*

To examine whether *StUBA2a/b* and *StUBA2c* have roles in the ABA response of guard cells, similar to *Arabidopsis* UBA2a and UBA2b, and VfAKIP1 (Bove *et al.*, 2008; Ng *et al.*, 2004), both genes were cloned under the control of a guard-cell-specific *pGC1* promoter (Yang *et al.*, 2008). To construct the final cassette of *pGC1:smGFP-StUBA2a/b* or -*StUBA2c*, first the *pGC1* promoter (−1140/+23) was PCR-amplified from genomic DNA from *Arabidopsis* Col-0 using gene-specific primers (Supplementary Table S1) and cloned into SacII/XhoI sites of the pORE-R2 binary vector (Coutu *et al.*, 2007). Subsequently, *smGFP*, *StUBA2a/b*, and *StUBA2c* were amplified using gene-specific primers (Supplementary Table S1). The *smGFP* PCR fragment was cloned into the XhoI/NotI site of the pORE-R2-pGC1 vector, followed by introduction of *StUBA2a/b* or *StUBA2c* to the Not1/SpeI or Not1/KpnI sites of the pORE-R2 pGC1:smGFP vector, respectively. Images of smGFP or smGFP-StUBA2s expressed in *Arabidopsis* were obtained using a FV500 confocal microscope (Olympus).

### *Agrobacterium tumefaciens*-mediated transient or stable transformation

The pORE-R2 binary vectors with *35S:StUBA2s* or *pGC1:smGFP-StUBA2s* constructs were electroporated into *Agrobacterium tumefaciens* strain C58C1, which was used to generate stable transgenic *Arabidopsis* plants by the floral dip method (Clough and Bent, 1998). Putative transgenic plants were screened on half-strength MS plates containing kanamycin. Kanamycin-resistant seedlings were transplanted to soil for the T2 seed set. T2 or T3 plants were used for phenotypic analysis. For the transient expression assay, *Agrobacterium* containing pORE-R2 binary vector harbouring target constructs, *35S:StUBA2a/b*, *35S:StUBA2a/b*, or *35S:GUS* as a vector control, was grown overnight in lysogeny broth media supplemented with 50mg L^−1^ of kanamycin and 150 μM acetosyringone. Agro-infiltration was performed on 1-month-old *N. tabacum* leaves following the method previously described (Kim *et al.*, 2008). The hypersensitive-like cell death phenotype was photographed 21 days after Agro-infiltration.

### PCR and gene expression analysis

RNA was extracted from *Arabidopsis* plants or from potato using the RNasy RNA extraction kit (Qiagen). The total RNA concentration was quantified by spectrophotometric measurement, and 1 or 2 µg of total RNA was used for cDNA synthesis using either Superscript III reverse transcriptase (Invitrogen) or a cDNA EcoDry Premix-Oligo dT Kit (Clontech). PCR was carried out in 20 μL reactions containing 1 μL of cDNA and 0.1 µM of gene-specific primers (Supplementary Table S1) using *Ex-Taq* DNA polymerase (TaKaRa) under the following conditions: an initial denaturation step at 95°C for 1min followed by 25 cycles of denaturation at 94°C for 30 s, annealing at 56°C for 30 s, polymerization at 72°C for 0.5–2min, and a final extension at 72°C for 7min. For real-time PCR, cDNA was diluted to a concentration of 1:10, and quantitative reverse transcriptase (qRT)-PCR was performed using SYBR Premix Ex Taq (TaKaRa). Actin was used as an internal control, and the data obtained were analysed with IQ5 software (Bio-Rad). The expression analysis of autophagy-associated genes (*Arabidopsis TOR*, *ATG8b-h*, *ATG9*, and *ATG18a*) was carried out using *Arabidopsis TOR* or *ATG* gene-specific primers (Supplementary Table S1).

### 3,3ʹ-Diaminobenzidine staining

To determine H_2_O_2_ accumulation, leaves of *35S:StUBA2s* transgenic lines and wild-type *Arabidopsis* were stained with 3,3ʹ-diaminobenzidine (DAB) solution according to a protocol described previously (Wohlgemuth *et al.*, 2002). Briefly, 1-month-old leaves from the lower position were immersed overnight in staining solution containing 1mg mL^−1^ of DAB and de-stained by soaking in 100% ethanol for 3h.

### Extraction of free SA

SA was extracted and analysed by gas chromatography-mass spectrometry (Ultra GC-Q/MS; Shimadzu Inc.) using a method described by Park *et al.* (2012). Three-week-old leaves (0.05g) were ground in liquid nitrogen and the powdered samples were extracted twice with 1mL of 90% methanol at 30°C for 10min. Supernatants were collected by centrifuging at 13 000rpm for 10min at 4°C and then mixed with 50 µL of 3,4,5-trimethoxycinnamic acid (100 µg mL^−1^) as an internal standard, followed by extraction twice with ethyl acetate. The ethyl acetate fraction was dried in a centrifugal concentrator (CVE-2000; Eyela). For derivatization of the dried extracts, 40 µL of *N*-(*tert*-butyldimethylsilyl)-*N*-methyltrifluoroacetamide containing 1% *tert*-butyldimethylchlorosilane (TBDMCS) and 40 µL of pyridine were added to the dried extracts, followed by incubation at 60°C for 30min at a mixing frequency of 1200rpm using a Thermomixer Comfort (model 5355; Eppendorf AG). Each derivatized sample (1 µL) was separated on a 30 m × 0.25mm internal diameter fused-silica capillary column coated with 0.25 µm CP-SIL 8 CB low bleed (Varian Inc.). The injector temperature was 230°C, and the flow rate of helium gas through the column was 1.0mL min^−1^. The temperature programme was set at 150°C and maintained at 150°C for 2min, followed by a 15°C min^−1^ oven temperature ramp to 320°C, which was held for 10min. The column effluent was later introduced into a QP2010 Ultra mass spectrometer (Shimadzu Inc.). The transfer line and the ion-source temperatures were 250°C and 200°C, respectively. The detected mass range was 85–700 *m/z*. The quantity of SA was calculated based on the ratio of the major fragment ion (*m/z* 309) of the *tert*-butyldimethylsilyl (TBDMS) derivative of SA and the corresponding fragment ion (*m/z* 295) of the internal standard.

## Results

### Cloning of potato *StUBA2a/b* and *StUBA2c*

**Fig. 1.**
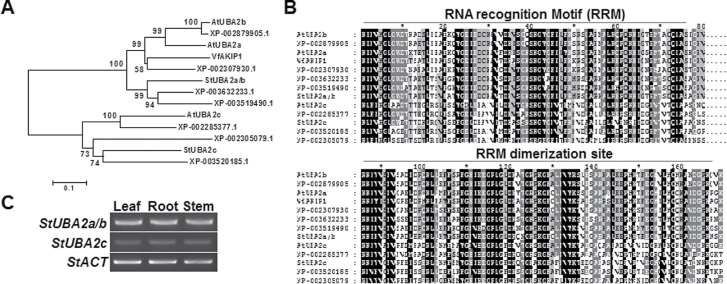
Phylogenetic analysis and comparison of potato StUBA2s and VfAKIP1 homologues in various plant species. (A). Phylogenetic tree of given VfAKIP1 homologues including two newly identified potato StUBA2s, generated using MEGA6 (Tamura *et al.*, 2013) and tree view programs (Page, 1996). Numbers on the tree denote percent homology from 2000 bootstrap replicates. (B) Comparison of conserved RRM domains among given VfAKIP1 homologues. (C) Expression patterns of *StUBA2a/b* and *StUBA2c* in potato leaf, root, and stem.

To identify VfAKIP1 homologs from *S. tuberosum*, amino acid sequences of VfAKIP1 and three *Arabidopsis* UBA2s were aligned against the TIGR potato EST database (http://plantta.jcvi.org) using BLAST. Two *AKIP1-like* EST sequences, TA24992_4113 and TA26496_4113, were obtained from the BLAST search. Amino acid sequences of TA24992_4113 showed higher sequence identity with VfAKIP1, and *Arabidopsis* UBA2a and UBA2b, while amino acid sequences of TA26496_4113 showed higher sequence identity with UBA2c. These EST sequences were used to clone 1577bp and 1349bp full-length cDNAs from potato cultivar ‘Atlantic’. The former was designated as *StUBA2a/b* and the latter as *StUBA2c*. Alignment of StUBA2s with VfAKIP1 and *Arabidopsis* UBA2s showed that StUBA2a/b had 42–43% amino acid sequence identity with UBA2a and UBA2b, and VfAKIP1, and that StUBA2c had 45% identity with UBA2c (Supplementary Table S2). Phylogenetic analysis using StUBA2s and various plant VfAKIP1 homologues showed distinctive separation between StUBA2a/b and StUBA2c. StUBA2a/b grouped with VfAKIP1, *Arabidopsis* UBA2a, and *Arabidopsis* UBA2b, while StUBA2c grouped with *Arabidopsis* UBA2c in a separate clade (Fig. 1A). The comparison of deduced amino acid sequences of the plant VfAKIP1 homologues including StUBA2s revealed that RRM domains are highly conserved in both StUBA2s (Fig. 1B; Supplementary Fig. S1). RT-PCR analysis showed that *StUBA2a/b* and *StUBA2c* expressed comparably in all tested tissues: leaf, root, and stem (Fig. 1C).

### Overexpression of StUBA2s induces early leaf senescence associated with hypersensitive-like cell death

**Fig. 2.**
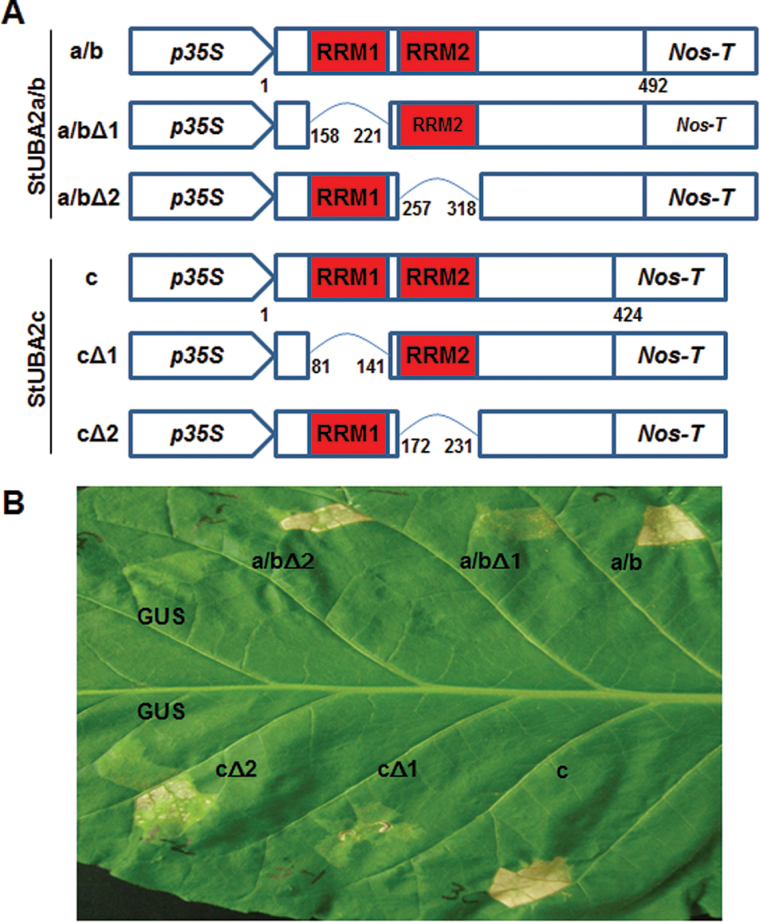
Transient expression of potato *StUBA2a/b* and *StUBA2c* induces hypersensitive-like cell death in *N. tabacum* leaves. (A) Schematics of *StUBA2a/b*, *StUBA2c*, and RRM deletion constructs in a modified *pORE-R2* vector carrying the *35S* promoter. (B) Hypersensitive-like cell death caused by StUBA2a/b and StUBA2c. All constructs were transiently expressed in tobacco leaves by Agro-infiltration. Hypersensitive-like cell death was induced by the transient expression of *35S:StUBA2a/b* (a/b), *35StUBA2a/bΔ2* (a/bΔ2), *35S:StUBA2c* (c), or *35S:StUBA2cΔ2* (cΔ2) but not by *35StUBA2a/bΔ1* (a/bΔ1) or *35S:StUBA2cΔ1* (cΔ1). A *35S:GUS* (GUS) construct was used as a control. Pictures were taken 20 days after Agro-infiltration (this figure is available in colour at JXB online).

It has previously been reported that the transient expression of *Arabidopsis* UBA2s induces hypersensitive-like cell death in *N. benthamiana* leaves, while their constitutive expression under the control of the *35S* promoter causes lethality at the young seedling stage (Kim *et al.*, 2008). Because StUBA2a/b and StUBA2c had high amino acid sequence identity to VfAKIP1 and *Arabidopsis* UBA2s, it was of interest to test whether overexpression of StUBA2s could induce the same hypersensitive-like cell death phenotype as *Arabidopsis* UBA2s. First, a transient expression assay of StUBA2s was carried out. For this test, pORE-R2 binary vectors containing *StUBA2s* under the control of a constitutive *35S* promoter (Fig. 2A) were introduced into *N. tabacum* leaves by Agro-infiltration. *N. tabacum* leaves started to show hypersensitive-like cell death symptoms within 1 week after Agro-infiltration, and symptoms were severely aggravated within 3 weeks (Fig. 2B), indicating that StUBA2s are most likely functional potato homologues of the *Arabidopsis* UBA2s. In parallel with full-length StUBA2a/b and StUBA2c, RRM-domain-deleted StUBA2s (Fig. 2A) were also expressed transiently to examine whether RRM domains play important roles in the induction of hypersensitive-like cell death. As a result, it was found that the first RRM domain in both StUBA2s is crucial for the induction of hypersensitive-like cell death (Fig. 2B).

**Fig. 3.**
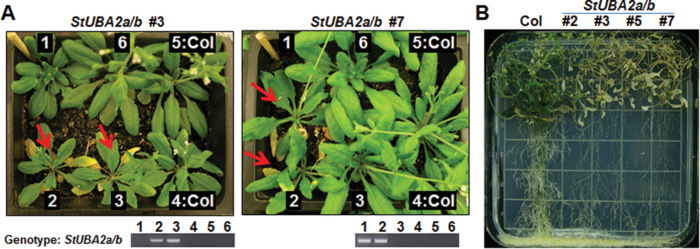
Early cell death/senescence of *Arabidopsis* plants correlates with presence of the *35S:StUBA2a/b* transgene. (A) Phenotype and genotype were examined for 1-month-old *35S:StUBA2a/b* T_2_ transgenic lines, #3 and #7. Six plants were planted in each pot, four plants randomly picked from each 2-week-old T2 segregating transgenic line and two untransformed *Arabidopsis* Col-0 plants, and numbered from 1 to 6 as shown. Early senescing phenotype was observed in PCR-confirmed transgenic plants as indicated by red arrows. Genomic DNA from individual plants was used for PCR-genotyping using *StUBA2a/b* gene-specific primers to detect the presence of transgene *StUBA2a/b*. (B) The senescing phenotype was re-examined in T3 *35S:StUBA2a/b* transgenic lines, and these lines exhibited early death of 3-month-old *35S:StUBA2a/b* plants on an MS plate (this figure is available in colour at JXB online).

**Fig. 4.**
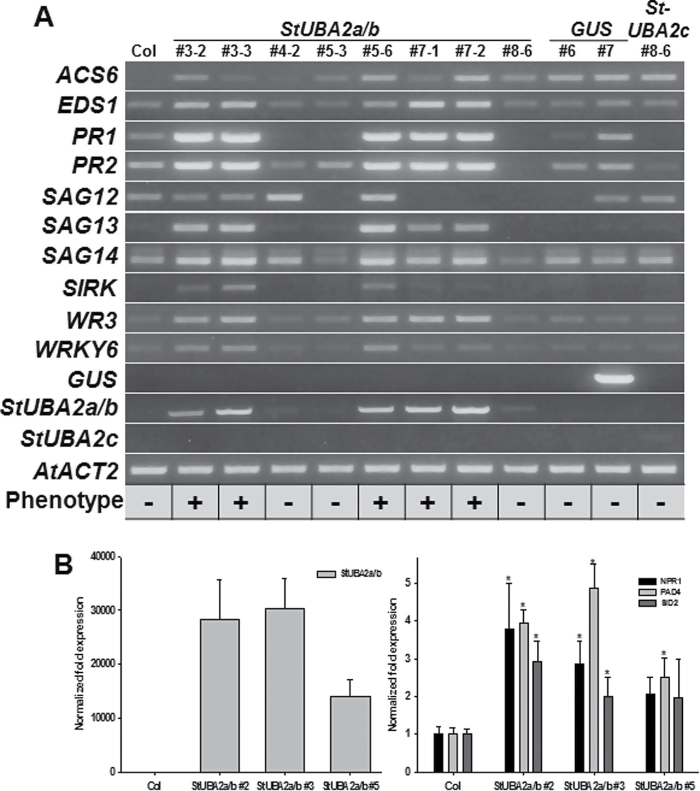
Overexpression of *StUBA2a/b* altered expression patterns of senescence- and defence-associated genes. (A) Transcript level of *StUBA2a/b* correlated with early senescing phenotype (‘Phenotype’), accompanied by expression changes of various genes. Total RNA from wild-type and T2 segregating *35S:StUBA2a/b* transgenic plants including those shown in Fig. 3A was isolated and used for RT-PCR analyses. (B) Elevated transcript levels of genes involved in SA signalling or biosynthesis in T3 *35S:StUBA2a/b* plants. qRT-PCR was carried out using *StUBA2a/b* gene-specific primers or primers specific to *NPR1*, *PAD3*, and *SID2* involved in SA signalling or biosynthesis (Supplementary Table S1). Transcript levels were normalized by transcript levels of *Arabidopsis ACT2*. Among *StUBA2a/b* transgenic lines, #2, #3, and #7 lines were heterozygous, and #5 was homozygous. Five-week-old rosette leaves were used for RNA extraction for qRT-PCR. T3 transgenic plants with kanamycin resistance were used for experiments unless otherwise stated except for those in Fig. 3A, Fig. 4A, and Supplementary Fig. S2B, where T2 plants were used. Asterisk indicates a significant difference at *P* < 0.05.

To further investigate the roles of StUBA2 proteins, stable *35S:StUBA2a/b* and *35S:StUBA2c* transgenic *Arabidopsis* plants were generated using the same binary vector as used for the transient expression assay. Unlike constitutive expression of *Arabidopsis UBA2s*, which terminated growth of transgenic *Arabidopsis* plants at the young seedling stage (Kim *et al.*, 2008), the overexpression of *StUBA2a/b* did not cause a lethal phenotype during early development, which made it possible to perform phenotypic analysis at later stages. Segregating T2 *StUBA2a/b* transgenic plants were planted in soil along with wild-type (Col) plants as control. Some of the T2 *StUBA2a/b* overexpressor lines started to show a yellowing phenotype in old leaves 6 weeks after transplanting (Fig. 3A). Genotyping of segregating *35S:StUBA2a/b #*3 and #7 T2 plants using *StUBA2a/b* specific primers showed that the presence of the transgene coincided with the leaf-yellowing phenotype (Fig. 3A). The senescing phenotype was re-examined in T3 *35S:StUBA2a/b* transgenic plants, in which it was found that the phenotype was aggravated with increasing plant age (Fig. 3B; Supplementary Fig. S2). The T2 transgenic plants shown in Fig. 3A were used for a survey of transcript levels of transgene, and defence- and senescence-associated genes as shown in Fig. 4A. The extent of the hypersensitive-like cell death in *35S:StUBA2a/b* transgenic lines coincided with the levels of transgene expression, which may explain why no such phenotype was detected in plants with low expression level of the *StUBA2c* transgene as summarized in ‘Phenotype’ (Fig. 4A). Except for T2 plants used in Fig. 3A and Fig. 4A, T3 transgenic lines were used for all phenotypic analyses.

In addition to *UBA2s* and *StUBA2s*, constitutive expression of another set of *Arabidopsis* RBPs, LARP1b and LARP1c, was recently reported to induce precocious leaf senescence in *Arabidopsis* (Zhang *et al.*, 2012). These gain-of-function mutants showed reduced chlorophyll content and hypersensitive-like cell death starting from leaves at a lower position, which is very similar to the phenotype observed in *StUBA2a/b* transgenic plants. Transcript level of the native *LARP1c* increased along with the progression of leaf senescence, and overexpression of *LARP1c* induced the expression of *SAG12* and *13*, suggesting that LARP1c and SAGs may be involved in plant senescence concomitantly (Zhang *et al.*, 2012). Therefore, an examination of whether overexpression of *StUBA2a/b* or *StUBA2c* induced the upregulation of *LARP1b* and *LARP1c* (Supplementary Fig. S3) was performed, but this was not the case. Also, leaf senescence induced by *StUBA2a/b* did not show any correlation with *SAG12* expression (Fig. 4A), implying that the expression of StUBA2a/b and *Arabidopsis* UBA2s induce leaf senescence through overlapping but non-identical mechanism(s) from *LARP1c*.

### Upregulation of stress- and defence-associated genes in StUBA2a/b *Arabidopsis*

**Fig. 5.**
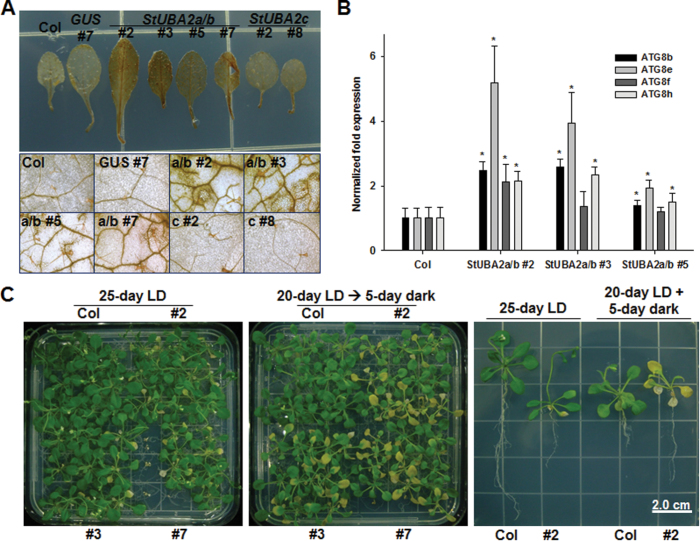
Early senescence of *35S:StUBA2a/b* transgenic plants is associated with H_2_O_2_ accumulation and the expression of autophagy-associated genes. (A) H_2_O_2_ accumulation in *35S:StUBA2a/b* transgenic plants grown on a half-strength MS plate for one month under permissive long-day light conditions (16h light, 22°C/12h dark, 20°C) at light intensity of 100 µmol m^−2^s^−1^. (B) Altered expression of autophagy-associated genes in *35S:StUBA2a/b* transgenic plants. qRT-PCR was carried out using *ATG8b*, *ATG8e*, *ATG8f*, and *ATG8h* gene-specific primers (Supplementary Table S1). Asterisk indicates a significant difference at *P* < 0.05. (C) Senescence of *35S:StUBA2a/b* transgenic plants was enhanced under darkness. Seeds of transgenic lines and wild type (Col) were sown on half-strength MS plates and grown for 25 days under the same permissive long-day light conditions as described above. For darkness, the plate was wrapped with aluminium foil on the 20^th^ day and otherwise maintained in the same conditions for 5 more days. Because *StUBA2a/b* #2, #3, and #7 seeds from T2 heterozygous parents were sown in kanamycin-free MS media in this experiment, some progeny (WT) from *StUBA2a/b* #2, #3, and #7 lines did not show the senescing phenotype.

Plants expressing StUBA2a/b show phenotypes similar to both age-dependent leaf senescence and the hypersensitive response. To better understand the pathways induced by StUBA2a/b expression, the transcript levels of genes associated with pathogen response, leaf senescence, SA, and autophagy were analysed (Figs. 4 and 5B; Supplementary Table S3). Leaf senescence is accompanied by changes in the expression of genes such as *SAGs* and defence-related genes. Consistently, leaf senescence caused by the overexpression of *Arabidopsis UBA2*s also induces the altered expression of various stress-responsive genes (Kim *et al.*, 2008), providing the plausible hypothesis that constitutive expression of *StUBA2a/b* also could alter the expression of such genes. *StUBA2a/b* transgenic lines #3, #5, and #7 exhibited altered expression of enhanced disease susceptibility 1 (*EDS1*), *PR1* and *PR2*, *SAG13*, *SAG14*, and *WOUND-RESPONSIVE 3* (*WR3*) among tested genes (Fig. 4A). Upregulation of these genes is consistent with previous findings concerning *Arabidopsis UBA2* genes, indicating that StUBA2a/b is most likely a UBA2a or UBA2b functional homologue in potato. Transcript levels of the genes *1-AMINOCYCLOPROPANE-1-CARBOXYLIC ACID SYNTHASE 6* (*ACS6*) and *WRKY6* were not consistent in two separate RT-PCR analyses, suggesting that the expression of these genes is not only influenced by the *StUBA2a/b* transgene, but also by other factors such as plant age.

Recently, it was reported that *Arabidopsis* RNA-binding protein-defence related 1 (*Arabidopsis* RBP-DR1), with three RRM domains, is involved in pathogen defence through SA signalling (Qi *et al.*, 2010). To examine whether StUBA2a/b also affects SA signalling, transcript levels of genes involved in SA biosynthesis and signalling (Lu, 2009; Ng *et al.*, 2011) were examined. *NPR1*, *PAD4*, and *SID2* were upregulated in *StUBA2a/b* transgenic lines and their transcript levels showed positive correlation with *StUBA2a/b* expression levels (Fig. 4B; Supplementary Fig. S4), indicating that *StUBA2a/b* expression may be involved in SA signalling and biosynthesis. To examine whether upregulation of SA signalling genes was due to or resulted in increased SA content, SA was measured by gas chromatography-mass spectrometry using a GCMS-PQ2010 Ultra (Shimadzu). SA content in *StUBA2a* transgenic lines was found to be significantly higher than that in wild-type plants (Supplementary Fig. S5).

### Correlation of hypersensitive-like cell death in StUBA2a/b plants with H_2_O_2_ accumulation and autophagy

Excessive ROS accumulation can damage plant cells and can cause necrosis either directly or indirectly via programmed cell death (Van Breusegem and Dat, 2006). To test whether hypersensitive-like cell death caused by StUBA2 expression is associated with ROS accumulation, 5-week-old rosette leaves of wild-type, *GUS* (vector control), *StUBA2a/b*, and *StUBA2c* transgenic lines were stained with DAB. H_2_O_2_ accumulation was detected in *StUBA2a/b* transgenic plants (Fig. 5A). Consistent with the hypersensitive-like cell death phenotype, *StUBA2c* transgenic plants expressing low levels of the transgene did not exhibit H_2_O_2_ accumulation. Because higher levels of H_2_O_2_ can cause severe oxidative damage in *Arabidopsis*, followed by induction of autophagy (Perez-Perez *et al.*, 2012), whether StUBA2a/b expression alters transcript levels of genes involved in autophagy was examined. RT-PCR and qRT-PCR were carried out to examine the expression of seven *Arabidopsis* autophagy-related genes: *TOR*, *ATG8* (*b*, *e*, *f*, and *h*), *ATG9*, and *ATG18a*. Four genes, *Arabidopsis ATG8b*, *e*, *h*, and *Arabidopsis ATG9* were upregulated in *StUBA2a/b* plants (Fig. 5B; Supplementary Fig. S6), indicating that *StUBA2a/b* expression plausibly induces autophagy through ROS accumulation. However, *Arabidopsis TOR*, a key negative regulator for the induction of autophagy, was not influenced by *StUBA2a/b* expression compared to wild type (Supplementary Fig. S6), suggesting that those genes with altered expression resulting from *StUBA2a/b* transgene expression may be regulated by other mechanisms independent from *Arabidopsis* TOR. Because the leaf senescence of *Arabidopsis* autophagy mutants is enhanced under darkness (Liu and Bassham, 2012), the response of *StUBA2a/b* transgenic plants to darkness was determined. Five days of darkness enhanced the yellowing symptom of *StUBA2a/b* plants (Fig. 5C), but no changes were detected in autophagosome formation compared to wild type (data not shown), as assayed by monodansylcadaverine staining of roots treated with 5-day darkness (Contento *et al.*, 2005; Liu and Bassham, 2010).

### Subcellular localization of StUBA2s

**Fig. 6.**
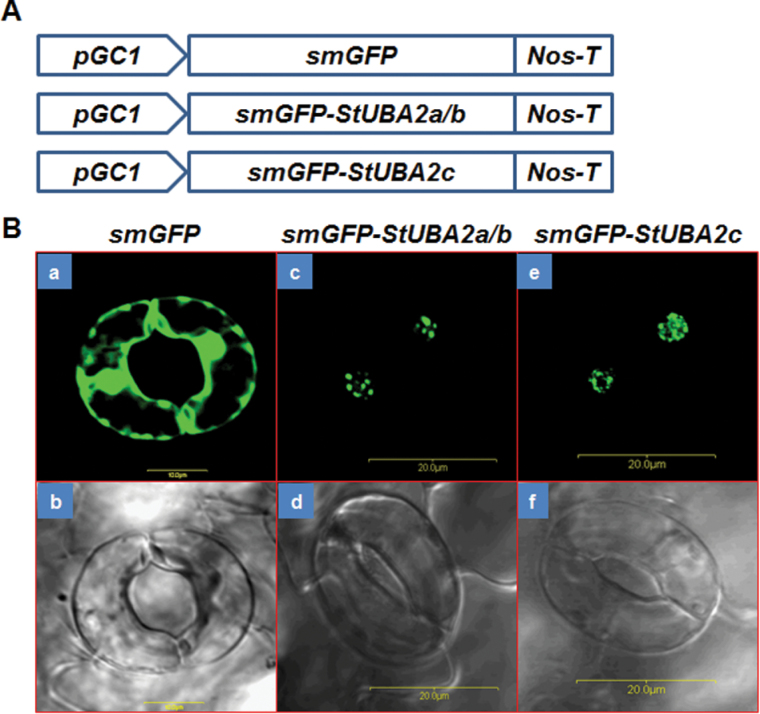
Localization of smGFP-StUBA2s in guard cells. (A) Diagram of constructs used for guard-cell-specific *StUBA2a/b* and *StUBA2c* expression in modified pORE-R3 vector. *pGC1*, guard-cell-specific promoter (Yang *et al.*, 2008); smGFP, green fluorescence protein cloned from pORE-R3. (B) Confocal images were taken from leaves of 2-week-old seedlings. (a) smGFP image; (b) bright field image of (a); (c) smGFP-StUBA2a/b image; (d) bright field image of (c); (e) smGFP-StUBA2c image; (f) bright field image of (e) (this figure is available in colour at JXB online).

In *V. faba*, VfAKIP1 is involved in ABA signalling in guard cells (Li *et al.*, 2002) and ABA induces rapid subnuclear relocalization of VfAKIP1, UBA2a, and UBA2b proteins into nuclear speckles (Li *et al.*, 2002; Ng *et al.*, 2004). To examine StUBA2 localization, *smGFP-StUBA2s* under the control of the *pGC1* promoter (Fig. 6A), a guard-cell-specific promoter (Yang *et al.*, 2008), were transformed into *Arabidopsis*. Nuclear speckles were observed in guard cells of both *pGC1:smGFP-StUBA2a/b* and *pGC1:smGFP*-*StUBA2c* transgenic plants (Fig. 6B). Because it was reported that exogenous ABA application can rearrange or enhance VfAKIP1-GFP subnuclear localization into nuclear speckles, leaves of wild-type and transgenic plants were submerged in ABA solution as described previously (Li *et al.*, 2002) and examined under a confocal microscope for smGFP-StUBA2s-marked nuclear speckles. However, no changes were observed in smGFP-StUBA2s localization after ABA treatment (Supplementary Fig. S7), similar to UBA2c-GFP fusion proteins (Bove *et al.*, 2008). These results suggest that StUBA2a/b and StUBA2c can reside in nuclear speckles, but that this localization is not affected by ABA, although it is difficult to exclude the possibility that this level of endogenous ABA may already be sufficient for StUBA2 proteins to form stable foci.

## Discussion

It has been reported that VfAKIP1 and *Arabidopsis* UBA2s are hnRNP-like RBPs involved in wounding, senescence (Bove *et al.*, 2008; Kim *et al.*, 2008), and ABA signalling in guard cells (Bove *et al.*, 2008; Li *et al.*, 2002; Ng *et al.*, 2004; Riera *et al.*, 2006). Despite described roles of VfAKIP1 and *Arabidopsis* UBA2s in stress and senescence, no other VfAKIP1 homologs have been identified and characterized from other plant species. In this study, the physiological roles of two VfAKIP1-like proteins from *S. tuberosum* ‘Atlantic’ were investigated, with a particular interest in leaf senescence. Controllable shoot senescence could confer an agronomic benefit. Potato growers routinely remove potato shoots prior to harvest to facilitate harvesting, improve setting of skin colour, and minimize disease infection from the shoots (Chen *et al.*, 2009). Therefore, timely potato shoot decay upon harvest could have a beneficial economic impact on potato farming, which may be achievable through altered expression of gene(s) related to plant senescence such as StUBA2s or *Arabidopsis* UBA2s.

### Leaf senescence of *35S:StUBA2a/b* plants was enhanced upon flowering

In agreement with the hypersensitive-like cell death and leaf senescence caused by overexpression of *Arabidopsis UBA2s* (Kim *et al.*, 2008), transient expression or stable expression of *StUBA2a/b* or *StUBA2c* under the constitutive *35S* promoter induced hypersensitive-like cell death in tobacco (Fig. 2B). *StUBA2a/b* transgenic *Arabidopsis* plants showed slight growth reduction, but did not exhibit lethality or severe growth arrest at a young seedling stage as was observed in *35S:UBA2s* transgenic *Arabidopsis* plants (Kim *et al.*, 2008). Rather, the leaf senescence of *StUBA2a/b* transgenic plants started to appear at a much later developmental stage than in *35S:UBA2s* plants, close to the time of flower initiation, and was aggravated upon flowering, suggesting that the mode of *StUBA2a/b* action may be closely related to the change of phase associated with flowering. The phenotype also apparently required a certain threshold in transcript/protein level to exert its role, as evidenced by RT-PCR results in which the transcript level of transgene *StUBA2a/b* correlated with the degree of the senescing phenotype (Fig. 4A). Also, older leaves of *StUBA2a/b* plants exhibited higher H_2_O_2_ accumulation than young leaves (Supplementary Fig. S8), implying that certain levels of ROS accumulation may be required for the initiation of hypersensitive-like cell death preceding leaf senescence in *StUBA2a/b* transgenic plants.

The *SAG12* gene is well known as a senescence-associated marker and its expression is tightly correlated with age-dependent leaf senescence/cell death (Kim *et al.*, 2009). Age-dependent leaf senescence of wild-type *Arabidopsis* plant ecotype ‘Col-0’ starts about 28 days after germination and requires the activation of ORESARA1 (ORE1) transcription factor (Kim *et al.*, 2009), of which transcript level is itself regulated by ETHYLENE INSENSITIVE 2 (EIN2). *ORE1* and *SAG12* expression show tight correlation with progression of aged-leaf senescence (Kim *et al.*, 2009). Aged-leaf senescence and *SAG12* expression were delayed in the *ore1* mutant and further delayed in an *ore1* and *ein2* double mutant (Kim *et al.*, 2009), indicating that *ORE1* and *SAG12* coincidently express in accordance with leaf senescence. Opposite to delayed age-dependent leaf senescence in *ore1* or *ein2* mutants, leaf senescence was accelerated in the *senescence-associated ubiquitin ligase1* (*saul1*) mutant, in which *ORE1* and *SAG12* started to accumulate much earlier than in wild type (Vogelmann *et al.*, 2012). Since the senescing phenotype of *StUBA2a/b* and *Arabidopsis UBA2* overexpressing plants did not correlate with *SAG12* expression, these results strongly suggest that leaf senescence induced by this family of hnRNPs may not be through the age-dependent leaf senescence pathway as also reported by Kim *et al.* (2008). Intriguingly, the transcript level of *ORE1* but not *EIN2* was upregulated in *StUBA2a/b* plants (Supplementary Fig. S9), implying that leaf senescence caused by StUBA2a/b and UBA2s likely is related to the upregulation of ORE1, but independent from ORE1-EIN2-SAG12-associated age-dependent leaf senescence mechanism(s). Therefore it would be interesting to examine whether cell death in *Arabidopsis* UBA2s and StUBA2a/b overexpression plants can be sustained in an *ore1* mutant background, in which leaf senescence is delayed.

### Do ROS production and autophagy correlate with hypersensitive-like cell death in *StUBA2a/b* transgenic plants?

*StUBA2a/b* expression in *Arabidopsis* caused H_2_O_2_ accumulation in leaves (Fig. 5A), and homolysis of H_2_O_2_ to 2OH^−^ can damage plant cells (Becana *et al.*, 1998), suggesting that the hypersensitive-like cell death phenotype of *StUBA2a/b* plants could be attributed to elevated H_2_O_2_. As one ROS, H_2_O_2_, is known to be involved in various signalling processes in plants and is generated from various sources, including as a by-product of reactions in chloroplasts, mitochondria, and peroxisomes (Tripathy and Oelmuller, 2012). Because ROS accumulation is harmful to cells, death or signalling depends on how cells regulate ROS homeostasis. Ironically, however, cell death is also an essential part of the life cycle by which multicellular organisms can recycle nutrients to maintain proper growth and development (Van Breusegem and Dat, 2006). ROS and autophagy have long been known to be associated with cell death, but recent studies reveal that they are also involved in signalling and acclimation under adverse stress conditions (Perez-Perez *et al.*, 2012). H_2_O_2_ accumulation activates autophagy, which is involved in the recycling of reusable molecules and damaged intracellular components or toxic molecules. *StUBA2a/b* expression in *Arabidopsis* altered the expression of autophagy-associated genes, *ATG8* (*ATG8b*, *e*, and *h*) and *ATG9* (Fig. 5B; Supplementary Fig. S6), implying that H_2_O_2_ resulting from *StUBA2a/b* expression indeed changed autophagy signalling by increasing the expression of *ATG* genes. It was reported that nitrogen remobilization in several *ATG* mutants including *atg5* was significantly reduced (Guiboileau *et al.*, 2012), indicating that autophagy plays important roles in nitrogen recycling and remobilization in plants. Nutrition deficiency also can induce ROS production in plants, which can activate autophagy. As shown in Fig 3A, early leaf senescence at the lower position in *StUBA2a/b* plants is similar to symptoms observed in *atg* mutants (Yoshimoto *et al.*, 2009; Guiboileau *et al.*, 2012), and three *ATG8* genes were upregulated in *StUBA2a/b* transgenic plants under normal conditions. Together, these results raise questions about whether *StUBA2s* are involved in autophagy-mediated regulation of nitrogen uptake or remobilization, which will be interesting topics for future studies.

### *StUBA2a/b* expression induced the expression of genes involved in senescence, defence, and SA signalling

Constitutive expression of *StUBA2a/b* in *Arabidopsis* increased the expression of various genes involved in defence and senescence, such as *PRs* and several *SAGs*, as shown in Fig. 4A, which is consistent with the results observed in transgenic *Arabidopsis* with inducible overexpression of *Arabidopsis UBA2s* (Kim *et al.*, 2008), as well as in transgenic tobacco expressing *LARP1c* (Zhang *et al.*, 2012). In addition, *StUBA2a/b* also elevated the transcript levels of *SID2*, *NPR1*, and *PAD4* involved in SA biosynthesis or signalling in *Arabidopsis* (Fig. 4B; Supplementary Fig. S4), suggesting that StUBA2a/b also affects SA signalling. *SID2* is upstream in the SA biosynthesis pathway where it catalyses conversion of chorismate to isochorismate (Chen *et al.*, 2009), and NPR1 and PAD4 can induce SA accumulation by feedback amplification (Lu, 2009; Ng *et al.*, 2011). It is well known that SA accumulation can induce hypersensitive-like cell death. Therefore, SA content may be elevated in *StUBA2a/b* plants, which would promote cell death, as was the case here (Supplementary Fig. S5).

### Nuclear speckles formed by smGFP-StUBA2s are not reorganized by exogenous ABA

Nuclear speckles are interchromatin granule clusters enriched in pre-mRNA splicing factors (Spector and Lamond, 2011). Nuclear speckles are believed to play crucial roles in gene expression as sites of pre-mRNA processing such as mRNA splicing (Reddy *et al.*, 2012). VfAKIP1 is relocalized to nuclear speckles by ABA treatment and also plays important roles in ABA-mediated stomatal regulation (Li *et al.*, 2002; Ng *et al.*, 2004). To examine *StUBA2* localization and ABA-response in guard cells, *Arabidopsis* transgenic plants were generated that constitutively expressed *smGFP-StUBA2s* in guard cells. Because it was shown that VfAKIP1 and *Arabidopsis* UBA2a and UBA2b fused to GFP relocalize to nuclear speckles following a few minutes of ABA treatment, localization of *smGFP-StUBA2s* with and without exogenous ABA application was compared. smGFP-StUBA2s fusion proteins were visualized as nuclear speckles (Fig. 6B). However, reorganization of nuclear speckles upon exogenous ABA application was not observed in *pGC1:smGFP-StUBA2s* plants (Supplementary Fig. S7). Also, the accelerated leaf senescence phenotype was not observed in *pGC1:smGFP-StUBA2s* plants.

In summary, potato RBP StUBA2a/b is a positive regulator of a leaf senescence, which likely is accelerated by hypersensitive-like cell death caused by H_2_O_2_ accumulation and the activation of SA signalling. This hypersensitive-like cell death/senescence phenotype occurs earlier than age-dependent leaf senescence. Furthermore, StUBA2a/b also induces genes involved in autophagy signalling that are related to nitrogen mobilization, which is yet to be examined. Thus, leaf senescence induced by potato RBP StUBA2a/b appears to arise from activation of components of several distinct cell-death pathways. Because timely leaf senescence upon potato harvest could confer a tremendous economic impact on potato farming, StUBA2s potentially can be considered for transgenic approaches that can induce potato shoot senescence at harvest.

## Supplementary data

Supplementary data can be found at *JXB* online.

Table S1. List of primers used in this study

Table S2. Identity and similarity among StUBA2s and other homologous proteins.

Table S3. Characteristics of genes used for expression assays.

Fig. S1. Alignment of full-length StUBA2s with homologous proteins in several plant species. (A) StUBA2a/b and its homologous proteins. (B) StUBA2c and its homologous proteins.

Fig. S2. Early leaf senescence of *35S:StUBA2a/b* plants grown aseptically in Magenta boxes, or in soil. (A). Senescing phenotype observed in 6-week-old T3 *35S:StUBA2a/b* plants in MS media. # numbers refer to independent transgenic lines. Each T3 transgenic line was germinated on an MS plate containing kanamycin and then kanamycin-resistant plants were transferred to antibiotic-free MS media. (B). Senescing phenotype observed in 2-month-old T2 transgenic plants grown in soil in a growth chamber. These plants are the same as those shown in Fig. 3A, but at a later developmental stage. Numbers indicate individual T2 plants from either *35S:StUBA2a/b* #3 or *35S:StUBA2a/b* #7.

Fig. S3. Transcript levels of *LARP1* RBP family are not altered by the overexpression of StUBA2a/b.

Fig. S4. Elevated transcript levels of genes involved in SA signalling or biosynthesis in T3 *35S:StUBA2a/b* plants. RT-PCR was carried out using gene specific primers (Supplementary Table S1). Among *StUBA2a/b* transgenic lines, #2, #3, and #7 lines were heterozygous, and #5 was homozygous. *StUBA2c* #2 and #8 and *GUS* #7 lines were homozygous. *GUS* #7 line was used as vector control.

Fig. S5. SA content in 3-week-old *35S*:*StUBA2a/b* and wild-type plants grown in MS media under 120 µmol^−1^ m^−2^ s^−1^ light with 16h/8h light/dark conditions. Representative ion chromatogram of SA extracted from (A) Col and (B) StUBA2a/b #3 as TBDMS derivatives separated on a 30 m × 0.25mm internal diameter fused-silica capillary column coated with 0.25 µm CP-SIL 8 CB low bleed. The upper trace was recorded in SIM mode (*m/z* 309, quantification ion of SA). Internal standard: 3,4,5-trimethoxycinnamic acid. (C) SA contents in the specified genotypes. Asterisk indicates a significant difference at *P* < 0.05.

Fig. S6. Altered expression of autophagy-associated genes in *35S:StUBA2a/b* transgenic plants. RT-PCR was carried out using autophagy-associated gene-specific primers (Supplementary Table S1).

Fig. S7. smGFP-StUBA2s localization was not changed by exogenous ABA application.

Fig. S8. H_2_O_2_ accumulation in young or old leaves of *35S:StUBA2a/b* transgenic plants.

Fig. S9. Age-dependent leaf senescence marker gene *ORE1* is upregulated by the overexpression of *StUBA2a/b*. # numbers refer to independent T3 lines. (A) RT-PCR analysis of *ORE1*, *SID2*, *AAO3*, and *EIN2* genes. (B) qRT-PCR analysis of *AAO3*, *SID2*, and *ORE1* genes. For both panels, transcript level of *Actin2* was used as the reference. Asterisk indicates a significant difference at *P* < 0.05.

Supplementary Data
