# Supplementary material for: Expression of potato RNA-binding proteins StUBA2a/b and StUBA2c induces hypersensitive-like cell death and early leaf senescence in Arabidopsis
Source: J Exp Bot. 2015 May 5;66(13):4023–33. doi: 10.1093/jxb/erv207 (PMC4473998; doi:10.1093/jxb/erv207)
Supplement: Supplementary Data [file supp_66_13_4023__index.html]

Expression of potato RNA-binding proteins StUBA2a/b and StUBA2c induces hypersensitive-like cell death and early leaf senescence in Arabidopsis — Expression of potato RNA-binding proteins StUBA2a/b and StUBA2c induces hypersensitive-like cell death and early leaf senescence in Arabidopsis — Supplementary Data 

# Expression of potato RNA-binding proteins StUBA2a/b and StUBA2c induces hypersensitive-like cell death and early leaf senescence in *Arabidopsis*

## Supplementary Data

Data files

**Files in this Data Supplement:**

- Supplementary Data - Supplementary Data
