# Supplementary material for: Expression of potato RNA-binding proteins StUBA2a/b and StUBA2c induces hypersensitive-like cell death and early leaf senescence in Arabidopsis
Source: J Exp Bot. 2015 May 5;66(13):4023–33. doi: 10.1093/jxb/erv207 (PMC4473998; doi:10.1093/jxb/erv207)
Supplement: Supplementary Data [file supp_erv207_jexbot143693_file001.pdf]

Supplementary data for:

Expression of potato RNA binding proteins, *StUBA2a/b* and *StUBA2c*, induces early senescence in *Arabidopsis*

Jong-Kuk Na, Jae-Kwang Kim, Dool-Yi Kim, Sarah M. Assmann

Supplementary Table S1. List of primers used in this study.

No	Primer ID	Primer sequences (5’ → 3’)	Comments
1	StUBA2a/b-F	CCAAATCAGAAAACCCTAATTTCA	Cloning to PCR™-Blunt II-TOPO
2	StUBA2a/b-R	ACTTGGAAGTAGAGCCATAGCATT	Cloning to PCR™-Blunt II-TOPO
3	StUBA2c-F	TCTTCTCTGAGCGAAAATGGA	Cloning to PCR™-Blunt II-TOPO
4	StUBA2c-R	TCAGGAAATCACCACAACCA	Cloning to PCR™-Blunt II-TOPO
5	pGC1-F-SacII	AAAACCGCGGATGGTTGCAACAGAGAGGATG	Cloning to pORE-R2 vector
6	pGC1-R-XhoI	AAAACTCGAGATTTCTTGAGTAGTGATTTTGAAG	Cloning to pORE-R2 vector
7	smGFP-F-XhoI	TTGTTCTCGAGTCTAGA ATGGCGAGTAAAGGAGAAGA	Cloning to pORE-R2 vector
8	smGFP-R-NotI	AAAGCGGCCGCTTTTGTATAGTTCATCCATGC	Cloning to pORE-R2 vector
9	StUBA2a/b-F-NotI	AAAGCGGCCGCATGGCGAAGAAGCGAAAAACC	Cloning to pORE-R2 vector
10	StUBA2a/b-R-KpnI	AAAGGTACCACTTGGAAGTAGAGCCATAGCATT	Cloning to pORE-R2 vector
11	StUBA2c-F-NotI	AAAGCGGCCGCATGGATCTAACAAAGAAACGC	Cloning to pORE-R2 vector
12	StUBA2c-R-SpeI	AAAACTAGTTCAGGAAATCACCACAACCAT	Cloning to pORE-R2 vector
13	StACT-F	TCCTCCATTGAAAAGAACTATG	RT-PCR analysis
14	StACT-R	CCAGACACTGTACTTTCTCTC	RT-PCR analysis
15	StUBA2a/b-RT-F	TGGAGGAAGGTAATGAAGAGGA	RT-PCR analysis
16	StUBA2a/b-RT-R	GGCAAGAAGTCATCCTTGTACC	RT-PCR analysis
17	StUBA2c-RT-F	TGAAACATTCACCAAAGACCAG	RT-PCR analysis
18	StUBA2c-RT-R	TTGGGCACCTTTAAAGAAAGAA	RT-PCR analysis
19	SID2-F	CAAATCTCAACCTCCGTCGT	RT-PCR analysis
20	SID2-R	CAACAGCGATCTTGCCATTA	RT-PCR analysis

21	PUB13-F	TCTTGCAAAACGAAATGCAG	RT-PCR analysis
22	PUB13-R	TGTTTCTAGGCGAGCCAGTT	RT-PCR analysis
23*	NPR1-F	TGCATCAGAAGCAACTTTGG	RT-PCR analysis
24*	NPR1-R	CCGAGTTCCACGGTTTTAGA	RT-PCR analysis
25*	PAD4-F	GCAAATTGCAACGGGAGTAT	RT-PCR analysis
26*	PAD4-R	AGGGCCAGAATTGTTCATTG	RT-PCR analysis
27*	AtTOR-F	TCAGTCAGGCGAAATCTACTCTACT	RT-PCR analysis
28*	AtTOR-R	TATCCTAGCAATGATTTGAGGTAGC	RT-PCR analysis
29*	AtATG18a-F	TCGCGTCGACTCCTTCAAATCATTCTTCCATG	RT-PCR analysis
30*	AtATG18a-R	TCGCTCTAGATTAGAAAACTGAAGGCGGTTT	RT-PCR analysis
31*	AtATG9-F	GTCGACATGAGCAGTGGGCATAAGGGTCCAAATG	RT-PCR analysis
32*	AtATG9-R	GGGCCCTCACCGTAATGTGGTGCTTGATGTTG	RT-PCR analysis
33*	AtATG8b-F	AGATCTATGGAGAAGAACTCCTTCAAGC	RT-PCR analysis
34*	AtATG8b-R	TCTAGATTAGCAGTAGAAAGATCCACCAAATGT	RT-PCR analysis
35*	AtATG8e-F	AGATCTATGAATAAAGGAAGCATCTTT	RT-PCR analysis
36*	AtATG8e-R	TCTAGATTAGATTGAAGAAGCAACGAA	RT-PCR analysis
37	AtATG8f-F	AGATCTATGGCAAAAAGCTCGTTCAAG	RT-PCR analysis
38	AtATG8f-R	TCTAGAAGCAAGAGGTCTCTATTATGGAGATCC	RT-PCR analysis
39	AtATG8h-F	AGATCTATGAAATCGTTCAAGGAACAATACAC	RT-PCR analysis
40	AtATG8h-R	TCTAGATCAACCAAAGGTTTTCTCACTGCT	RT-PCR analysis
41	ORE1-F	CAGCCGGTTTACCTTCGTTA	RT-PCR analysis
42	ORE1-R	CCAAACGCAATCCAATTCTT	RT-PCR analysis
43	EIN2-F	CTTGGCTTCATCGTGCTACA	RT-PCR analysis
44	EIN2-R	ACCCCAGAAATCCCAAAAAC	RT-PCR analysis
45	AAO3-F	GTGAGGCGGTTTTTGTTGAT	RT-PCR analysis
46	AAO3-R	TCTTTGACCTGCACATCGAG	RT-PCR analysis
47	EDS16-F	ATGAGATTCAGCCTCGCTGT	RT-PCR analysis
48	EDS16-R	TGATGGATCTCCAATCGTCA	RT-PCR analysis
49	SAG12-F	GGATGTCCCGGTTAATGATG	RT-PCR analysis

50	SAG12-R	TCCACTTTCTCCCCATTTTG	RT-PCR analysis
51	LARP1a-F	GCCGATGATCGAGAATTGAT	RT-PCR analysis
52	LARP1a-R	GTTTTGAATGACGGCCAGAT	RT-PCR analysis
53	LARP1b-F	ATGCAGGCTCGAGTGTTTCT	RT-PCR analysis
54	LARP1b-R	TTTGGTTGCCATGCTCATAA	RT-PCR analysis
55	LARP1c-F	TGCTTCTGTATCCGTTGCTG	RT-PCR analysis
56	LARP1c-R	GCATTTGCAATTCCCTGAGT	RT-PCR analysis
57**	SAG13_F	TGTAATGGCTACAAATCTCGAGTC	RT-PCR analysis
58**	SAG13_R	CTAGTCTGCCGTCAAATTGGTAAT	RT-PCR analysis
59**	SAG14_F	AGGACTACGATGTTGGTGATGATA	RT-PCR analysis
60**	SAG14_R	GAGTGTGACTCAAAAGAGAGCAAC	RT-PCR analysis
61**	SIRK_F	ATTTATCTTGAGCTGGGAAGAGAG	RT-PCR analysis
62**	SIRK_R	GGCATACATATTATTCAGCAACCA	RT-PCR analysis
63**	WRKY6_F	AACTGAGTCCAACAAAATTCAGAAG	RT-PCR analysis
64**	WRKY6_R	GTGGTTCGTACCATTGATCATAGA	RT-PCR analysis
65**	PR1_F	ATCGTCTTTGTAGCTCTTGTAGGTG	RT-PCR analysis
66**	PR1_R	TGATACATCCTGCATATGATGCTC	RT-PCR analysis
67**	PR2_F	CTTACTTCAGCTACATGGGAGACA	RT-PCR analysis
68**	PR2_R	CAAGTTCCCAATTTTTAAATACGC	RT-PCR analysis
69**	PR5_F	ATGGCAAATATCTCCAGTATTCACA	RT-PCR analysis
70**	EDR1_F	AGATGAATACAAGCCAAAGTGTCA	RT-PCR analysis
71**	EDS1_R	AGCGTAATCCACCACTTTCTAAAC	RT-PCR analysis
72**	ACS6_F	GTAATCGAGGAGATCGAAGATTGTA	RT-PCR analysis
73**	ACS6_R	TACTCTGCCAACACTTCTTCTTCTT	RT-PCR analysis
74**	WR3_F	ACTTCTCATATGCTCACTGATCCA	RT-PCR analysis
75**	WR3_R	GAAAGAAGAAGTGTGCAACAAGAC	RT-PCR analysis
76	StUBA2a/b-dRRM1-R	TTCTTCT GGTGTGCCGGATCTTTATCA	StUBA2a/b RRM1 deletion
77	StUBA2a/b-dRRM1-F	GCACACC AGAAGAAGATTGGTACAAGGAT	StUBA2a/b RRM1 deletion
78	StUBA2a/b-dRRM2-R	TATGTGG CCTCTGTGTGTACTCCGAAAC	StUBA2a/b RRM2 deletion

79	StUBA2a/b-dRRM2-F	ACAGAGG CCACATAAGACCTTTGAAGGG	StUBA2a/b RRM2 deletion
80	StUBA2c-dRRM1-R	TGGGCAC GCGGTTAGATACGTCGGCGT	StUBA2c RRM1 deletion
81	StUBA2c-dRRM1-F	TAACCGC GTGCCCAACAAGATAATTGATG	StUBA2c RRM1 deletion
82	StUBA2c-dRRM2-R	GATCAAC CCTAAGCGCAACATCAGAAGA	StUBA2c RRM2 deletion
83	StUBA2c-dRRM2-F	GCTTAGG GTTGATCCCGTGAAGACAGTA	StUBA2c RRM2 deletion

* These primers were previously used by Liu and Bassham (2010).

** These primers were previously used by Kim et al (2008).

Reference

Liu Y, Bassham DC. 2010. TOR is a negative regulator of autophagy in Arabidopsis thaliana. PloS one 5: e11883

Kim CY, Bove J, Assmann SM. 2008. Overexpression of wound-responsive RNA-binding proteins induces leaf senescence and hypersensitive-like cell death. *New Phytologist* **180**, 57-70.

Supplementary Table S2. Identity and similarity among StUBA2s and other homologous proteins.

	**1**	**2**	**3**	**4**	**5**	**6**	**7**	**8**	**9**	**10**	**11**	**12**	**13**
**1. AtUBA2b**	**<u>451</u>**	91%	67%	46%	53%	46%	42%	42%	24%	24%	24%	24%	24%
**2. XP_002879905.1**	414	**<u>447</u>**	66%	45%	53%	46%	40%	42%	24%	25%	24%	24%	25%
**3. AtUBA2a**	325	321	**<u>478</u>**	47%	54%	47%	42%	43%	23%	24%	22%	24%	23%
**4. VfAKIP1**	250	242	259	**<u>515</u>**	57%	45%	40%	43%	23%	25%	23%	22%	23%
**5. XP_002307930.1**	263	260	271	307	**<u>474</u>**	50%	47%	46%	25%	25%	23%	24%	24%
**6. XP_003632233.1**	232	235	243	248	255	**<u>478</u>**	64%	58%	24%	23%	23%	23%	25%
**7. XP_003519490.1**	218	213	222	224	248	329	**<u>496</u>**	55%	24%	23%	21%	22%	23%
**8. <u>StUBA2a/b</u>**	221	221	228	242	244	296	283	**<u>492</u>**	24%	23%	21%	23%	23%
**9. AtUBA2c**	121	123	121	125	131	128	132	130	**<u>404</u>**	60%	46%	45%	42%
**10. XP_002285377.1**	131	130	132	139	138	131	133	132	275	**<u>449</u>**	43%	45%	39%
**11. XP_002305079.1**	119	120	115	125	122	121	117	113	191	198	**<u>384</u>**	45%	44%
**12. <u>StUBA2c</u>**	124	122	126	123	127	123	122	124	199	212	196	**<u>424</u>**	52%
**13. XP_003520185.1**	120	122	118	123	121	128	124	123	180	184	184	226	**<u>384</u>**

* Bold numbers with underline denote amino acid length of the corresponding protein.

Top lines with percentage denote aligned sequence identity and bottom lines indicate identical sequence length.

Supplementary Table S3. Characteristics of genes used for expression assays.

Locus ID	Gene/protein	Full name	Senescence type	Defense (cell death)	Pathway	Regulation by StUBA2a/b
At4g35890	LARP1c	La Related protein 1c	Age-dependent senescence	N/A	Senescence	no effect
At5g45890	SAG12	Senescence-Associated gene 12	Age-dependent senescence	N/A	Senescence	no effect
At2g29350	SAG13	Senescence-Associated gene 13	Age-dependent senescence	N/A	Senescence	upregulated
At1g62300	WRKY6	WRKY transcription factor 6	Age-dependent senescence	Plant defense	Senescence/Pathogen defense	slightly upregulated
At5g66100	LARP1b	La Related protein 1b	Age-dependent senescence	N/A	Senescence	no effect
At5g39610	ORE1	Oresara 1	Age-dependent senescence	N/A	Senescence	upregulated
At2g27150	AAO3	Abscisic aldehyde oxidase 3	Age-dependent senescence	N/A	ABA biosynthesis	slightly upregulated
At5g03280	EIN2	Ethylene insensitive 2	Age-dependent senescence	N/A	Ethylene signaling/stress response	no effect
At1g50030	AtTOR	Target of rapamycin	Accelerated senescence	N/A	Autophagy	no effect
At4g04620	AtATG8b	Autophagy 8B	Accelerated senescence	N/A	Autophagy	upregulated
At2g45170	AtATG8e	Autophagy 8E	Accelerated senescence	N/A	Autophagy	upregulated
At4g16520	AtATG8f	Autophagy 8F	Accelerated senescence	N/A	Autophagy	no effect
At3g15580	AtATG8h	Autophagy 8H	Accelerated senescence	N/A	Autophagy	slightly upregulated
At3g62770	AtATG18a	Autophagy 18A	Accelerated senescence	N/A	Autophagy/stress response	no effect
At2g31260	AtATG9	Autophagy 9	Accelerated senescence	Plant defense	Autophagy	slightly upregulated
At4g11280	ACS6	1-aminocyclopropane-1-carboxylate synthase 6	Ethylene-induced senescence	N/A	Ethylene biosynthesis	upregulated

At2g19190	SIRK	Senescence-induced receptor-like kinase	Leaf senescence	N/A	Senescence	upregulated
At5g50200	WR3	Wound-Responsive 3	Leaf senescence	Plant defense	Wounding response	upregulated
At3g48090	EDS1	Enhanced disease susceptibility 1	N/A	SA-mediated plant defense	SA signaling/Stress response	no effect
At2g14610	PR1	pathogenesis-related protein 1	N/A	Plant defense	Systemic acquired resistance	upregulated
At3g57260	PR2	pathogenesis-related protein 2	N/A	Plant defense	Systemic acquired resistance	upregulated
At5g20230	SAG14	Senescence-Associated gene 14	N/A	N/A	N/A	slightly upregulated
At5g21160	LARP1a	La Related protein 1a	N/A	N/A	N/A	no effect
At1g74710	SID2/EDS16	Salicylic acid induction deficient 2	N/A	SA-mediated plant defense	SA signaling/SA biosynthesis	slightly upregulated
At1g64280	NPR1	Nonexpressor of PR genes 1	SA-mediated leaf senescence	SA-mediated plant defense	SA signaling	upregulated
At3g52430	PAD4	Phytoalexin deficient 4	SA-mediated leaf senescence	Plant defense	SA signaling/SA accumulation	upregulated
At3g46510	PUB13	Plant U-Box 13	SA-mediated leaf senescence	SA-mediated plant defense	SA signaling	no effect

Supplementary Figures

A.

```
                         *        20         *        40         *        60
StUBA2a/b    : --MAKKRKTRAS--------EPSQPVEEPVEIKQEPEVETAQEEEYENDAVEVEEEEAVHED :  52
XP_003519490 : MPMAKKRKLRSSNPEPTKPVEPQQQNHETVELDQQQPIPEPTLEDSNTMAIDEPKHEQEEEP :  62
XP_003632233 : --MAKKRKLRSSEPESSKAPEQEEQPQPTQEEDDQTKFEPIQEDE------DQPQHMAEDEP :  54
AtUBA2b      : ------------------------------MTKKRKLESESNETSEPTEKQQQCEKEDPE :  31
XP_002879905 : ------------------------------MAKKRKLESESNETSEPTEK-QLQCEKEDPE :  30
AtUBA2a      : ------------------------------MTKKRKLEGEESNEAEEPSQKLKQTPEEEQQ :  31
VfAKIP1      : -----------MCDSQSETLVTTFVSREQQTMVRKRKLVAKSSQPEEPPLKQHHSQPQPDPE :  51
XP_002307930 : ------------------------------MARKRKLDSKSTETAEEPPKKQQQQEEP-PK :  30

                       *        80         *       100         *       120
StUBA2a/b    : EEEQDPEEEDEEGGDDEEEEEMPGSGNDAVEDDNEKSVNEQEQVNGGPEVKMEEGNEE--D : 112
XP_003519490 : QNAESEQEEEEEEEEDEQQEEQEPETREGQPGPETLEAANQAEANGSNEGNNEEEEEEDLT : 124
XP_003632233 : KHEAQGEEEEEEEGEGEEEEEEGDQEGDVDASNEAVADEVPADANG------DGVEEDEGD : 110
AtUBA2b      : IRNVDNQRDDDEQVVEQDTLKEMHEEEAKGEDNIEAETSS------------GSGNQGNEDD :  81
XP_002879905 : FRNDDKQRDHDEEVVG----EDALKEEAKGEDNTEAETSS------------GSANRGNGDD :  76
AtUBA2a      : LVIKNQDNQGDVEEVEYEEVEEEQEEEVEDDDDEDDGDENEDQTDGNRIEAAATSGSGNQED :  93
VfAKIP1      : PEPYIPTEEVEEEYEEVEEEYEEVEEIEVEEEEEEEEEEED---------GGEQAQGGEYLE : 104
XP_002307930 : EEPQEEEVEVEEEEEEYEEVEEEEEDNEDDPDGETNQNAQ---------ISAVENLNDDDD :  83

                     *       140         *       160         *       180
StUBA2a/b    : LEDEPLENLLEPFTKDQLTALIKEALAKYPDFKENIQKLADKDPAHRKIFVHGLGWDTTAET : 174
XP_003519490 : LEEEPVEKLLEPFTKEQLHSLVTQAVDMFPEFVDSVRRIADVDPAHRKIFVHGLGWDATAET : 186
XP_003632233 : VEDEEIEKLLEPFSKEQLLALLREAVEKYPDFVDSVRRLADADPAHRKIFVHGLGWDTTAET : 172
AtUBA2b      : DEEEPIEDLLEPFSKDQLLILLKEAAERHRDVANRIRIVADEDLVHRKIFVHGLGWDTKADS : 143
XP_002879905 : DEEEPIEDLLEPFSKDQLLILLKEAAERHSDVANRIRIVADEDSVHRKIFVHGLGWDTKAET : 138
AtUBA2a      : DDDEPIQDLLEPFSKEQVLSLLKEAAEKHVDVANRIREVADEDPVHRKIFVHGLGWDTKTET : 155
VfAKIP1      : EDDEPIKDLVEPFTKEQIATLLCEAAAKHRDVADRIRKIADGDASHRKIFVHGLGWDTTSAT : 166
XP_002307930 : EDEEPIEKLLEPFGKDQLINLLREAADGHRDVADKIRQVADQDPVHRKIFVHGLGWDTNAEA : 145

                   *       200         *       220         *       240
StUBA2a/b    : LTSVFATYGEIEDCKAVTDKVSGKSKGYGFILFKHRSGARKALKEPQKKIGTRMTSCQLASA : 236
XP_003519490 : LTSVFGKYGEIEDCKAVTDKVSGKSKGYAFILFKHRDDARKALKHPQKKIGNRTTSCQLASA : 248
XP_003632233 : LTSVFGKYGEIEDCKAVSDKISGKSKGYAFILFKHRSGARKALKQPQKKIGNRMTSCQLASA : 234
AtUBA2b      : LIDAFKQYGEIEDCKCVVDKVSGQSKGYGFILFKSRSGARNALKQPQKKIGTRMTACQLASI : 205
XP_002879905 : LIEAFKQYGEIEDCKCVVDKISGQSKGYGFILFKSRSGARNALKQPQKKIGTRMTACQLASI : 200
AtUBA2a      : LIEAFKQYGEIEDCKAVFDKISGKSKGYGFILYKSRSGARNALKQPQKKIGSRMTACQLASK : 217
VfAKIP1      : LINAFSQYGEIEDCKAVTDKASGKSKGYGFILFKKRSGARNALKEPQKKIGNRMTACQLASI : 228
XP_002307930 : LINAFKPYGEIEDCKAVCDKVTGKSKGYGFILFKKRSGARNALKEPQKKIGNRMAACQLAST : 207

                 *       260         *       280         *       300         *
StUBA2a/b    : GPVPAPPPTT---------AAPPVSEYTQRKIFVSNVAADLEPQKLLEYFSKFGEVEEGPLG : 289
XP_003519490 : GPVPAPPPS-----------VTPVSEYTQRKIFVSNVSAEIDPQKLLEFFKQFGEVEDGPLG : 299
XP_003632233 : GPVPAPPPV-----------VPPVSEYTQRKIFVSNVSSEIDPQKLLEFFAKFGEIEEGPLG : 285
AtUBA2b      : GPVQG------NPVVAPAQHFNP--ENVQRKIYVSNVSADIDPQKLLEFFSRFGEIEEGPLG : 259
XP_002879905 : GPVQG------TPVVAPAQHFNP--ENVQRKIYVSNVSADLDPQKLLEFFSRFGEIEEGPLG : 254
AtUBA2a      : GPVFGGAPIAAAAVSAPAQHSNS--EHTQKKIYVSNVGAELDPQKLLMFFSKFGEIEEGPLG : 277
VfAKIP1      : GPVAQTPQP-------AVQLVQPGSEYTQRKIYISNVGPELDPHKLFAYFSRFGEIEEGPLG : 283
XP_002307930 : GPVPQSSAGPSGPIAAAAQTQQPVSEYTLRKIYVSNVGANLDPQKLMSFFSEFGEIEEGPLG : 269
```

```
                  320         *        340         *        360         *
StUBA2a/b    : LDKQNGKPKGFCLFVYKTVEGARKALEEPHKTFEGHTLHCQKAIDGPKHSKNFHQQQQPQSQ : 351
XP_003519490 : LDKNTGKPKGFALFVYKSVESAKKALEEPNKNYEGHTLYCQKAVDGPKGSKGYHHQQHSHS- : 360
XP_003632233 : LDKSTGKPKGFALFVYKSIESSRRALEEPHKIFEGHTLHCQKAIDGPKPNKPFH-------- : 339
AtUBA2b      : LDKATGRPKGFALFVYRSLESAKKALEEPHKTFEGHVLHCHKANDGPKQVKQHQHNHN---- : 317
XP_002879905 : LDKVTGRPKGFALFVYRSLESAKKALEEPHKTFEGHVLHCHKANDGPKQVKQHQHNHN---- : 312
AtUBA2a      : LDKYTGRPKGFCLFVYKSSESAKRALEEPHKTFEGHILHCQKAIDGPKPGKQQQHHHN---- : 335
VfAKIP1      : LDKATGKPKGFCLFVYKSAESARRSLEEPHKEFEGHILHCQRAIDGPKAGKTQHPQPQQLQH : 345
XP_002307930 : LDKATGKPKGFCLFVYKTVESAKKALEEPHKSFEGHILHCQKAVDGPKHAKSQKPPQQ---- : 327

                380         *        400         *        420         *
StUBA2a/b    : QH------YPQHHHHNQQQHYYQHPAKKGKYSGSSAGAGSAGHLMAPSGPAPVGYNP---AV : 404
XP_003519490 : -----------HHHHHHHQPHYQRKEKNKYSSGGGGPSHGSGHLMAPSGSAVGGFNPG-VPA : 410
XP_003632233 : ------------HHHHQHQPHYPSRKDKSKYSANAAG---PGHLMAPSGPSMTLNPG---VA : 383
AtUBA2b      : -------------SHNQNS-RYQRNDNNGYGAPG-----GHGHFIAGNNQ----------AV : 350
XP_002879905 : -------------SHNQNS-RYQRNDSNGYGAPG-----GHGHFIAGNNQ----------AA : 345
AtUBA2a      : -------------PHAYNNPRYQRNDNNGYGPPG-----GHGHLMAGNPAGMGG-----PTA : 374
VfAKIP1      : VNSLNQFNQLNQLNPATQRTQFQRNDNVGYVGGSSVAVSQPGHLMAPAGPTIGYNQAAASAA : 407
XP_002307930 : ------------HHNMQSSHYQRNDGGGYVASG----GRGGHLMAPAAAGAGIGFNQSAAA : 372

              440         *        460         *        480         *
StUBA2a/b    : AAVT----PALGQALTALLATQGAGLGIGNLLGGFG----------GPVNHQGVQPVVNNAT : 452
XP_003519490 : QGLN----PVLGQALTALIN-QGAGLGLGNLLGGLGG---------APVNQGGPPAPYANQP : 458
XP_003632233 : PALN----PALGQALTALLASQGAGLGLTNLLGGLGG---------APVNQAMPPAAHGMQG : 432
AtUBA2b      : QAFN----PAIGQALTALLASQGAGLGLNQAFGQALLGTLGTAS---PGAVGGMPSGYGTQA : 405
XP_002879905 : QAFN----PAIGQALTALLASQGAGLGLNQAFGQALLGTLGTAS---PGAVGGMPSSYGTQA : 400
AtUBA2a      : QVIN----PAIGQALTALLASQGAGLAFNPAIGQALLGSLGTAAGVNPGNGVGMPTGYGTQA : 432
VfAKIP1      : QGLNPVLTPALGQALTALLASQGATLG-LSDLLGSLGTSNALNHGGVPAAGHGVQSGYSAQP : 468
XP_002307930 : PALN----PALGQALTALLATQGAGLGGLTNLLGTLGSAAAVTQGGVPSAAHGMQGAYGNQA : 430

            500         *        520         *        540         *
StUBA2a/b    : GYG---------AQGGYGAQPHMQYQNPQMQGGARPQ-------GGGAPYSGYGGH : 492
XP_003519490 : AMG---------YGNQPAMQPGYQNPQMGQSSGVRPHP------GAGAPYMGH--- : 496
XP_003632233 : MQGGYGNQGAGSYGSQPGMQGGYQNPQMGQASGGRPQ-------QGGAPYMGH--- : 478
AtUBA2b      : NISPGVYPGYGA---QAGYQGGYQTQQPGQGGAGRGQH---GAG-YGGPYMGR--- : 451
XP_002879905 : NITPGVYPGYAA---QAGFQGGYQTQQPGQGSAGRGQH---GVG-YGGPYMGQM-- : 447
AtUBA2a      : -MAPGTMPGYGT---QPGLQGGYQTPQPGQGGTSRGQH---GVGPYGTPYMGH--- : 478
VfAKIP1      : TISPSVMGAYGNSVPQVGLQVQYPNQQIGQGGSGRGQYGGA------ASYMGH--- : 515
XP_002307930 : SISPGVIGAYAN---QGAMQGGYPNQQMGQGGSGRGQHGQY------TPYMGH--- : 474
```

B.

```
                          *        20         *        40         *        60
AtUBA2c      : MDMMKKRKLDENGNGLNTNGGG------------TIGPTRLSPQDARKIIERFTTDQLLDLLQEAIV :  55
XP_002285377 : MDATKKRKMDENGLVSTDGDS----------------LTKLAPEDARKIIEPFGHDQLLEILQNAAV :  51
StUBA2c      : MDLTKKRKADENGGAYPVSN------ELVTTAAAPPALAVLSPEDIDKILETFTKDQCVSILRNAAL :  61
XP_003520185 : MEFPKKRKTQENGDTTTDSPPPQSTEEAPLAATPAPASPLASEDIRKILQPFSQEQLLDLLQSASL :  67
XP_002305079 : MEFIKKRKAEENGISIPTGTSP------------PSPITPLTPEEIRKILEPFTKDQLLDILQSATL :  55

                 *        80         *       100         *       120         *
AtUBA2c      : RHPDVLESVRLTADSDISQRKLFIRGLAADTTTEGLRSLFSSYGDLEEAIVILDKVTGKSKGYGFVT : 122
XP_002285377 : RHLDVLDAIRSIADRDLTQRKLFIRGLGWDTTTEGLRQLFSAYGELEEAVVILDKVTAKSKGYGFVT : 118
StUBA2c      : RYADVLEGLRAVADADVSNRKLFVRGLGWETTTDKLRQVFSEFGELDEAVVITDKASSRSKGYGFVT : 128
XP_003520185 : RHPDVLDAVRAVADCDSTLRKLFVRGLAGETTTETLRSVFSGFGELDEAIVIMDKATGRSKGYGFVV : 134
XP_002305079 : HHSDILNSVRSVADGDISLRKLFIRGLSSETTSDTLRILFSSFGELEEAIVIHDKNTGKSKGFGFIT : 122

                140         *       160         *       180         *       200
AtUBA2c      : FMHVDGALLALKEPSKKIDGRVTVTQLAASGNQGTGS--QIADISMRKIYVANVPFDMPADRLLNHF : 187
XP_002285377 : FKHVDGALLALKEPSKKIDGRMTVTQLAASGLSGPASNASAVDVSMRKIYVANVPVDMPADRLLAHF : 185
StUBA2c      : FKHVDAAILSLKVPNKIIDGRVTVTQLAAGNSGNSQ---SSDVALRKIYVGNVPFEISSEKLLNHF : 192
XP_003520185 : FRHVDGAILALKDPSKKIDGRMTVTQLAAGGPGGG------DVSLRKVFVGNVPFEISSERLLDEF : 195
XP_002305079 : FKHVDSAMLSIREPSKKIDGRITVTQLASNNS-------STGDVSLRKVYVGNVPFEITSERLLGFF : 182

                      *       220         *       240         *       260
AtUBA2c      : MAYGDVEEGPLGFDKVTGKSRGFALFVYKTAEGAQAALADPVKVIDGKHLNCKLAVDGKKGGK---- : 250
XP_002285377 : SSYGEIEEGPLGFDKQTGKSRGFALFVYKTAEAAQAALVDPTKMIDGRQLVCKLAIEGKKGKT---- : 248
StUBA2c      : SMYGEIEEGPLGFDKQTGKAKGFAFFVYKTEDGARASLVDPVKTVEGHQVLCKLATDNKKGKPQNMG : 259
XP_003520185 : LKFGEVEEGPLGFDKSSGKSRGFAFFVYKTEEGARASLVEPLKTIEGHQVICKLAVDNKKTKP---- : 258
XP_002305079 : SMYGEIEEGPLGFDKSTGKSKGFAFLIYKSEDGAKAAIADPMKNIDGHQVVCKLAVDNKRVNKTS-- : 247

                 *       280         *       300         *       320         *
AtUBA2c      : -PGMPQAQD---GGSGHGHVHGEGMGMVRPAGP----YGAAGGISAYGGYS---------------- : 293
XP_002285377 : -GGPAQAPTGVPGEAGHGDGLGVPPQGSMPGSLGSQYPGGPGGISAYGGFS---------------- : 298
StUBA2c      : HGGAPGMNAGPGGMPGDDRVGSMPGSNYGVPVSGLGPYSGFSGGPGPGMQQPPPQPGMVAHQNPHLN : 326
XP_003520185 : -----------FGTDQHQHQQQHPQQQMQQQVSMMVPQYGGYGGNGYGMQQ---------------- : 298
XP_002305079 : -----------QGGANAQNSQPLAHPSFPQPQPQNSIGGVTGSLQNYGSASN--------------- : 288

                340         *       360         *       380         *       400
AtUBA2c      : ---GGPP----AHHMNSTHSSMGVG-----------------------------------SAGYGGH : 318
XP_002285377 : ---GGPPPLGHHHPLNSSLPSSLGGGPGLSSQVPSSLGGSAGYGAGLGGPYGSAHYGGPNSAGYSGL : 362
StUBA2c      : SAIGGPG-------------------------------------------------YGNQGPGSF : 342
XP_003520185 : QQHQVPP-------------------------------------------------YNNQVPVAG : 314
XP_002305079 : ------------------------------------------------------SYQLNTSLTS : 298

                      *       420         *       440         *       460
AtUBA2c      : YGGYGGPGGTGVYGGLGGGYGGPGTGSGQYRMPPSSMPG-GGGYPESGHYGLSSSAGYPGQHHQAVG : 384
XP_002285377 : GGAGGLGGAAGALGVAGGALGVAGGGSSLYRLPPSTVGMPSSGGYPDSGHYSLSSSSAYPSQIHQPAG : 429
StUBA2c      : TGGAGGYAGAGGYAGAGGYGGSSGDYSGYRMPQSSAGMPSSGGYPDGGNYGLQ-----SSYPSQVPQ : 404
XP_003520185 : GGGYGHRYGNSQMAGP-----VSGDYGARVPPNS------GGGYPDGSQYGFP-----PSS-GHPPQ : 364
XP_002305079 : SGYNGAYRVPPYVGAGPNDGGLNNAGASMYRMPQSTVGTGSGVYPDAGSYALSQ----QQQHQQPSS : 361

                 *       480         *
AtUBA2c      : TSPVPRVPH--GGMYPNGPPNY- : 404
XP_002285377 : TSPVPRVPP--GGMYPSGPPYY- : 449
StUBA2c      : PGAGSRVPP--GGMYQGMPPYY- : 424
XP_003520185 : PRVPMARPP--GGMYQGAPPYY- : 384
XP_002305079 : MPLPPRFPHGAGGMYQGMPPPYY : 384
```

Supplementary Figure S1. Alignment of full-length potato StUBA2s with homologous proteins in several plant species. **A**. StUBA2a/b and its homologous proteins. **B**. StUBA2c and its homologous proteins.

A

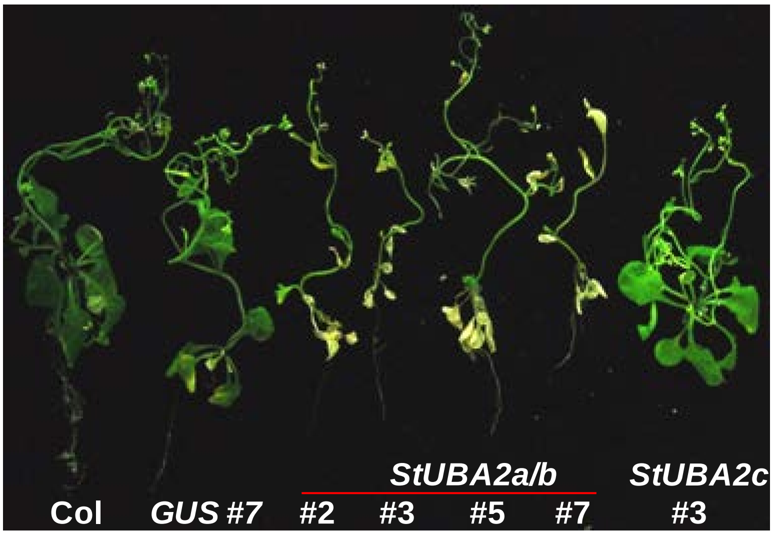

B

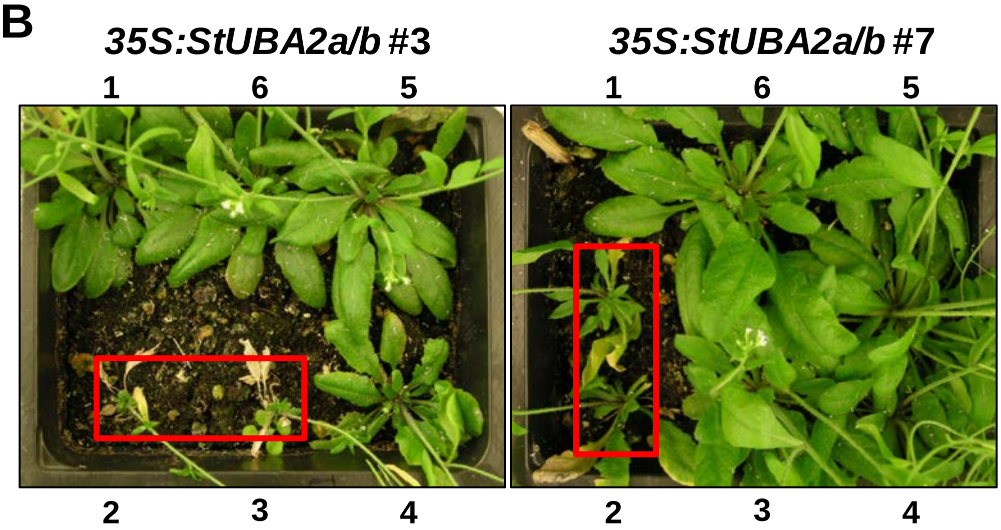

Supplementary Figure S2. Early leaf senescence of *35S:StUBA2a/b* plants grown aseptically in magenta boxes, or in soil. **A**. Senescing phenotype observed in six-week-old T3 *35S:StUBA2a/b* plants in MS media. # numbers refer independent transgenic lines. Each T3 transgenic line was germinated in MS plate containing kanamycin and then kanamycin-resistant plants were transferred to antibiotic-free MS media. **B**. Senescing phenotype observed only in two-month-old T2 transgenic plants grown in soil at growth chamber. These plants are the same as those shown in Fig. 3A, but at a later developmental stage. Numbers indicate individual T2 plants from either *35S:StUBA2a/b* #3 or *35S:StUBA2a/b* #7.

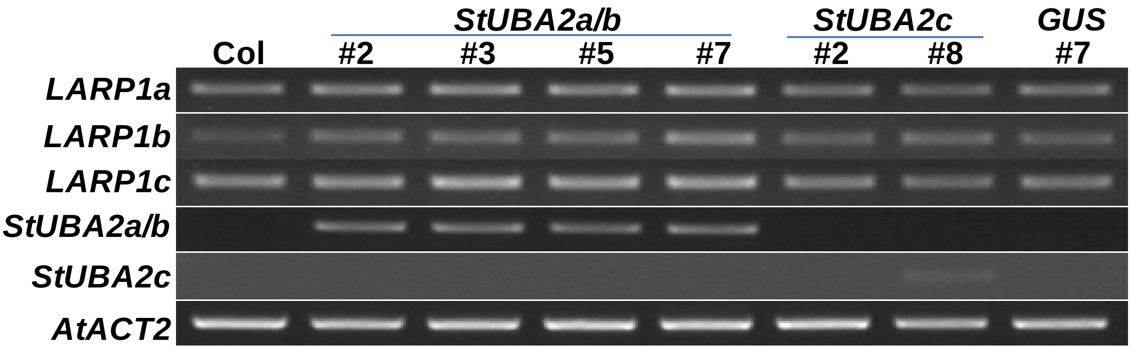

Supplementary Figure S3. Transcript levels of *LARP1* RBP family are not altered by the overexpression of StUBA2a/b.

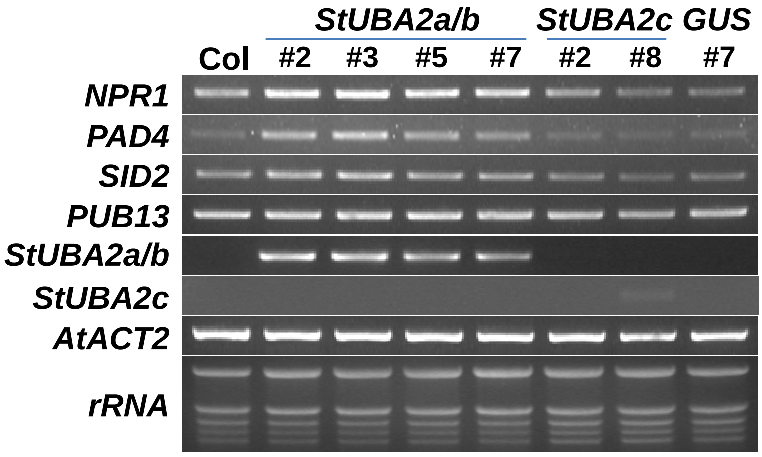

Supplementary Figure S4. Elevated transcript levels of genes involved in SA signaling or biosynthesis in T3 *35S:StUBA2a/b* plants. RT-PCR was carried out using gene specific primers (Supplementary Table S1). Among *StUBA2a/b* transgenic lines, #2, #3, and #7 lines were heterozygous, and #5 was homozygous. *StUBA2c* #2 and #8 and *GUS* #7 lines were homozygous. *GUS* #7 line was used as vector control.

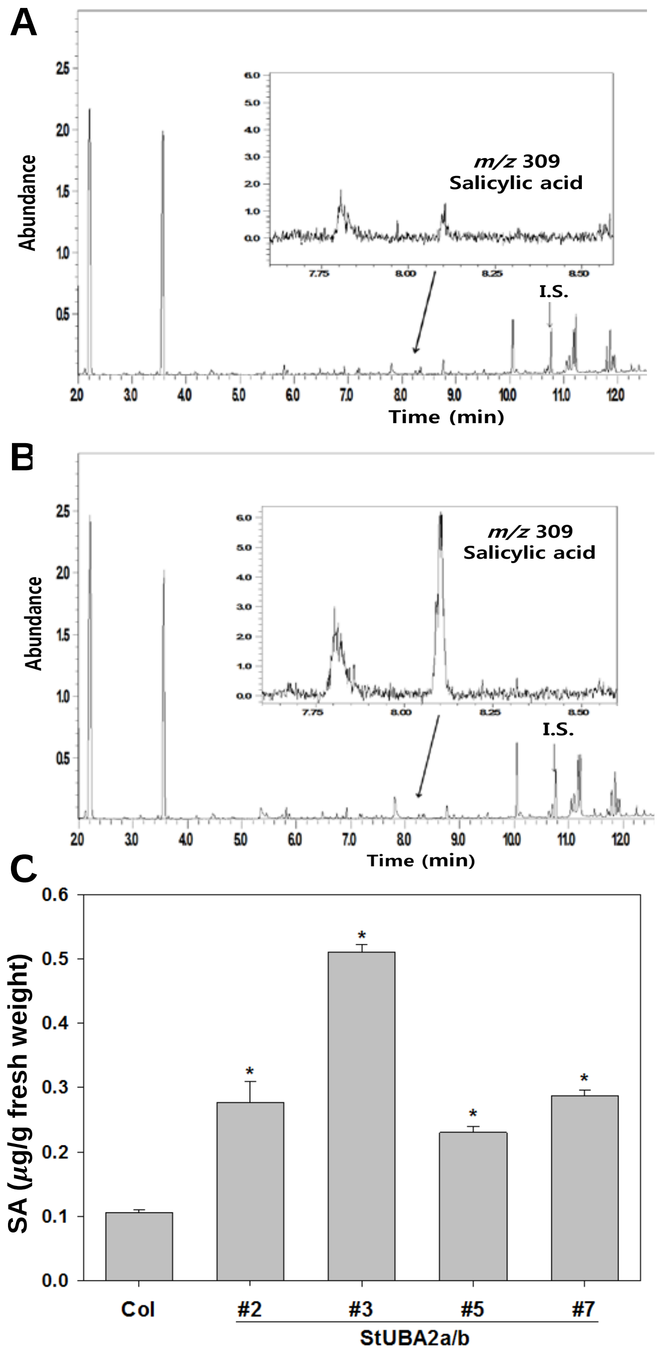

Supplementary Figure S5. SA content in three-week-old *35S*:*StUBA2a/b* and wild type plants grown in MS media under 120 μmol^{-1} m^{-2} light with 16/8 light/dark conditions. Representative total ion chromatogram of SA extracted from Col (**A**) and StUBA2a/b #3 (**B**) as TBDMS derivatives separated on a 30 m × 0.25 mm i.d. fused-silica capillary column coated with 0.25-μm CP-SIL 8 CB low bleed. The upper trace was recorded in SIM mode (*m/z* 309, quantification ion of SA). Internal Standard (I.S.) 3,4,5-trimethoxycinnamic acid. **C.** SA contents in the given genotypes. Asterisk indicates a significant difference at $P < 0.05$.

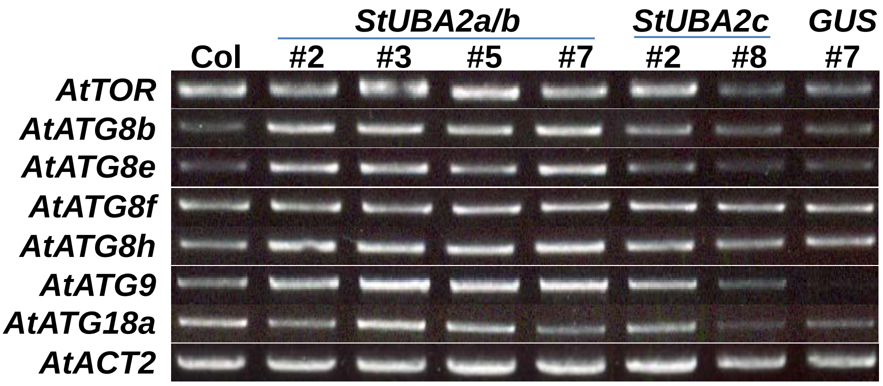

Supplementary Figure S6. Altered expression of autophagy-associated genes in *35S:StUBA2a/b* transgenic plants. RT-PCR was carried out using autophagy-associated gene specific primers (Supplementary Table S1).

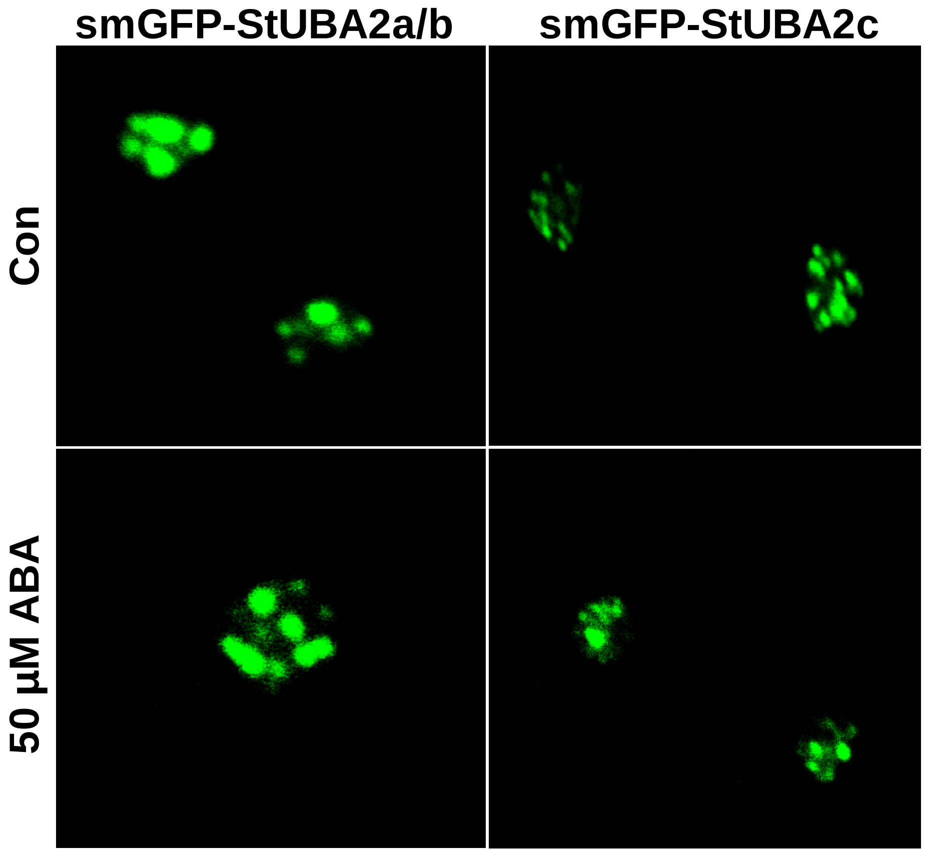

Supplementary Figure S7. smGFP-StUBA2s did not relocalize with exogenous ABA application.

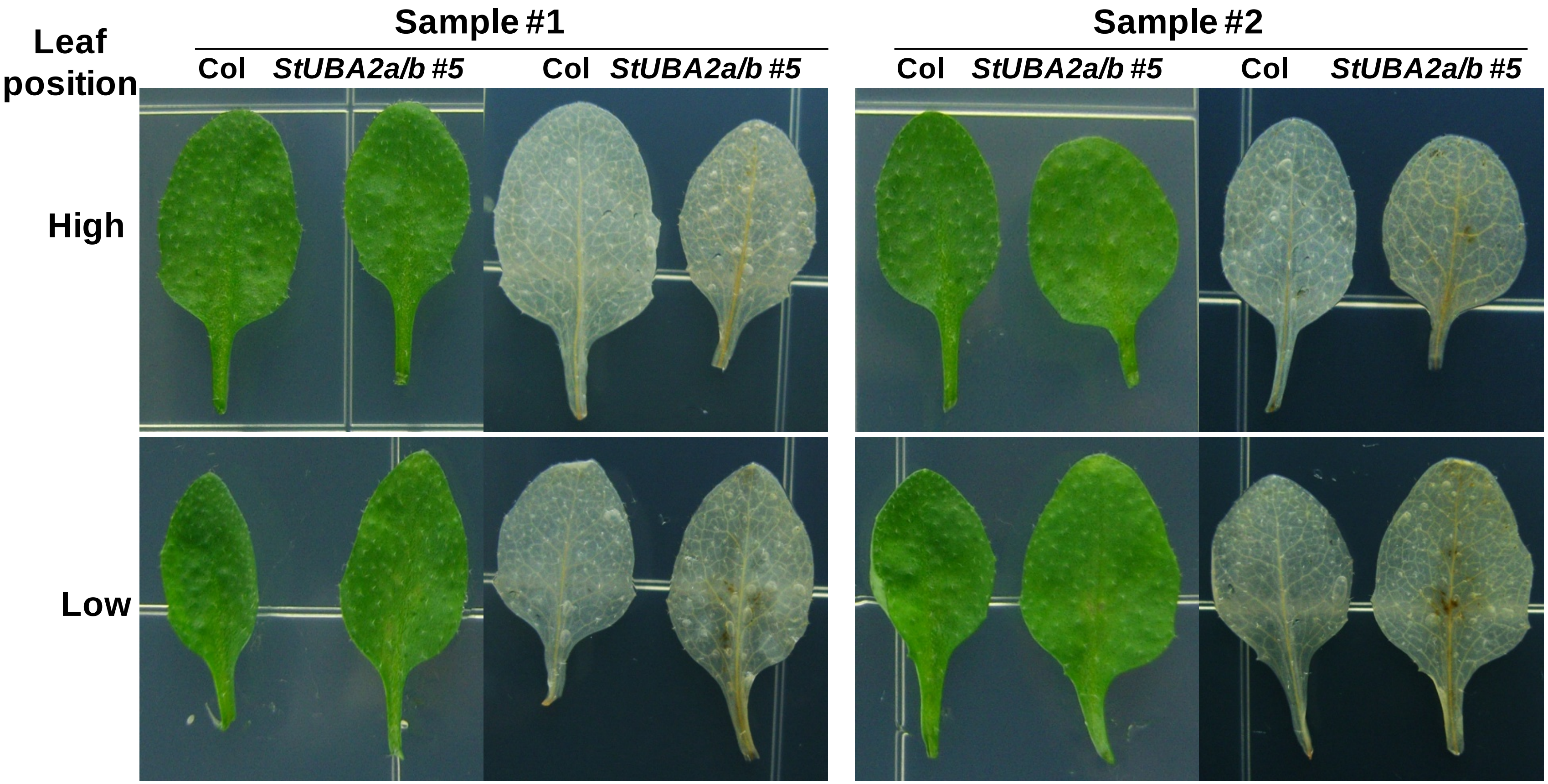

Supplementary Figure S8. H_2O_2 accumulation in the young or old leaves of *35S:StUBA2a/b* transgenic plants.

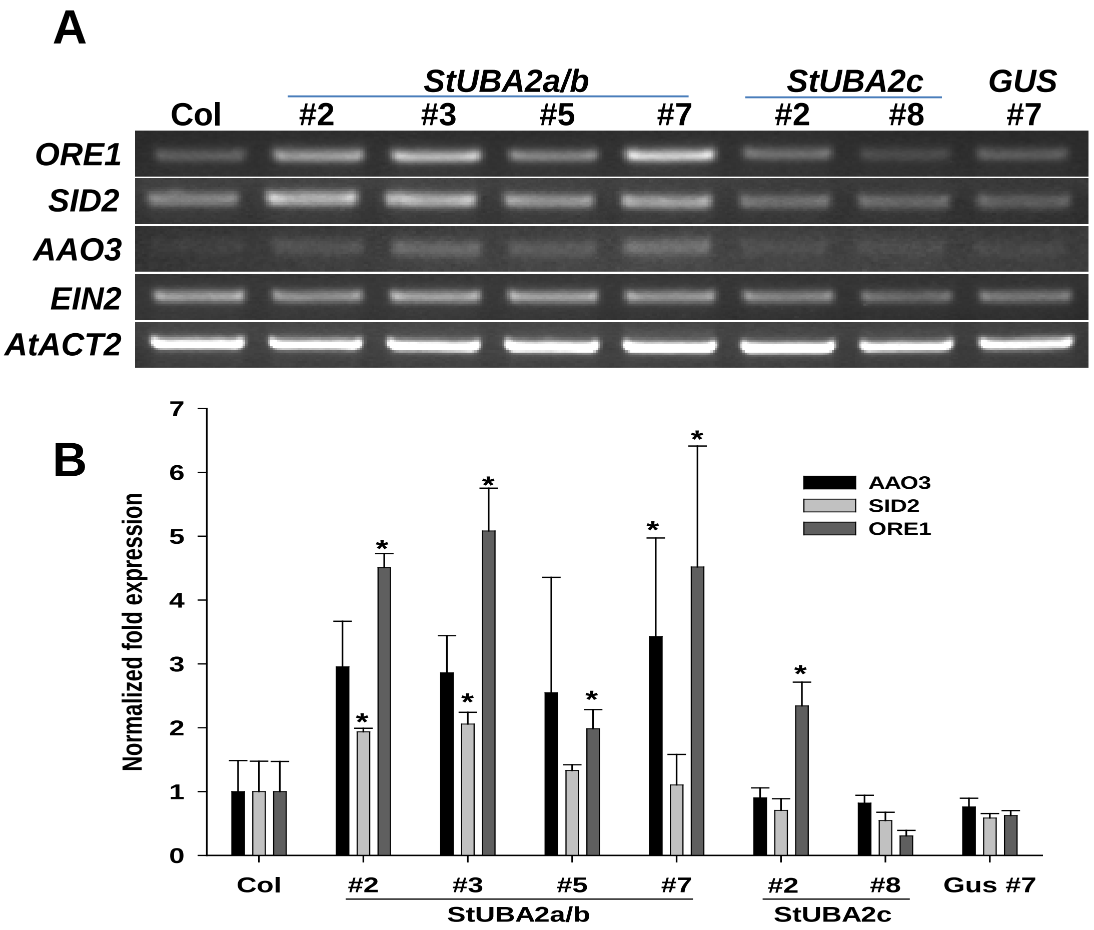

Supplementary Figure S9. Age-dependent leaf senescence marker gene *ORE1* is upregulated by the overexpression of *StUBA2a/b*. # numbers refer to independent T3 lines. **A**. RT-PCR analysis of *ORE1*, *SID2*, *AAO3*, and *EIN2* genes. **B**. qRT-PCR analysis of *AAO3*, *SID2*, and *ORE1* gene. Transcript level of *Actin2* gene was used as reference. Asterisk indicates a significant difference at $P < 0.05$.
